# A peptidomimetic with a chiral switch is an inhibitor of epidermal growth factor receptor heterodimerization

**DOI:** 10.18632/oncotarget.19013

**Published:** 2017-07-05

**Authors:** Shanthi P. Kanthala, Yong-Yu Liu, Sitanshu Singh, Rushikesh Sable, Sandeep Pallerla, Seetharama D. Jois

**Affiliations:** ^1^ Basic Pharmaceutical Sciences, School of Pharmacy, University of Louisiana at Monroe, Monroe LA 71201, USA

**Keywords:** HER2, protein-protein interaction, breast cancer, peptidomimetic, dual inhibitor

## Abstract

Among different types of EGFR dimers, EGFR-HER2 and HER2-HER3 are well known in different types of cancers. Targeting dimerization of EGFR will have a significant impact on cancer therapies. A symmetric peptidomimetic was designed to inhibit the protein-protein interaction of EGFR. The peptidomimetic (Cyclo(1,10)PpR (*R*) Anapa-FDDF-(*R*)-Anapa)R, compound 18) was shown to exhibit antiproliferative activity with an IC_50_ of 194 nM in HER2-expressing breast cancer cell lines and 18 nM in lung cancer cell lines. The peptidomimetic has a Pro-Pro sequence in the structure to stabilize the β-turn and a β-amino acid, amino napthyl propionic acid. To investigate the effect of the chirality of β-amino acid on the structure of the peptide and its antiproliferative activity, diastereoisomers of compound 18 were designed and synthesized. Structure-activity relationships of these compounds indicated that there is a chiral switch at β-amino acid in the designed compound. The peptidomimetic with R configuration at β-amino acid and with a L-Pro-D-Pro sequence was the most active compound (18). Using enzyme complement fragmentation assay and proximity ligation assay, we show that compound 18 inhibits HER2:HER3 and EGFR:HER2 dimerization. Surface plasmon resonance studies suggested that compound 18 binds to the HER2 extracellular domain and in particular to domain IV. The anticancer activity of compound 18 was evaluated using a xenograft model of breast cancer in mice; compound 18 suppressed the tumor growth in mice compared to control. Compound 18 was also shown to have a synergistic effect with erlotinib on EGFR mutated lung cancer cell lines.

## INTRODUCTION

Epidermal growth factor receptors (EGFR) play an important role in cell growth and differentiation in normal physiology. Dysregulation of signaling of these receptors are known to play a major role in the development of breast cancer, as well as lung and ovarian cancers. Thus, targeting EGFR can have a significant impact on the breast, lung, and ovarian cancer therapies [1–3]. The human epidermal growth factor receptor (HER) family of receptor tyrosine kinases plays an important role in cell growth and differentiation [4, 5]. The receptor system consists of four homologous family members—HER1–4. Binding of a ligand to the extracellular domains of these receptors induces a conformational change in the receptor structure. This conformational change sends signaling through the intracellular region via a transmembrane helix, a cytoplasmic kinase domain, and a regulatory region [5]. Dysregulation of homo-/heterodimerization processes of these receptors or overexpression of receptors plays a key role in tumor progression [6]. Amongst the several possible dimers of these receptors (EGFR-EGFR, EGFR-HER2, HER2-HER3, and HER2-HER4) implicated in cancer [7]. EGFR-HER2 and HER2-HER3 are known to play a significant role in the development and progression of different types of cancer. EGFR mutations seem to play a major role in breast and lung cancers. Studies related to breast and non-small cell lung cancers (NSCLC) have shown a link between HER2 expression and poor prognosis in patients with these cancers [8, 9]. Subjects with EGFR and HER2 mutations in the kinase domain, and treated with tyrosine kinase inhibitors (TKI) invariably develop resistance [10]. Further, it has also been shown that the overall survival rate is lower in patients who have high levels of EGFR and HER2 expression [2, 11–13]. Thus, targeting both EGFR and HER2 simultaneously may be an advantageous treatment approach in patients with tumors that overexpress EGFR and HER2.

**Figure 1:**
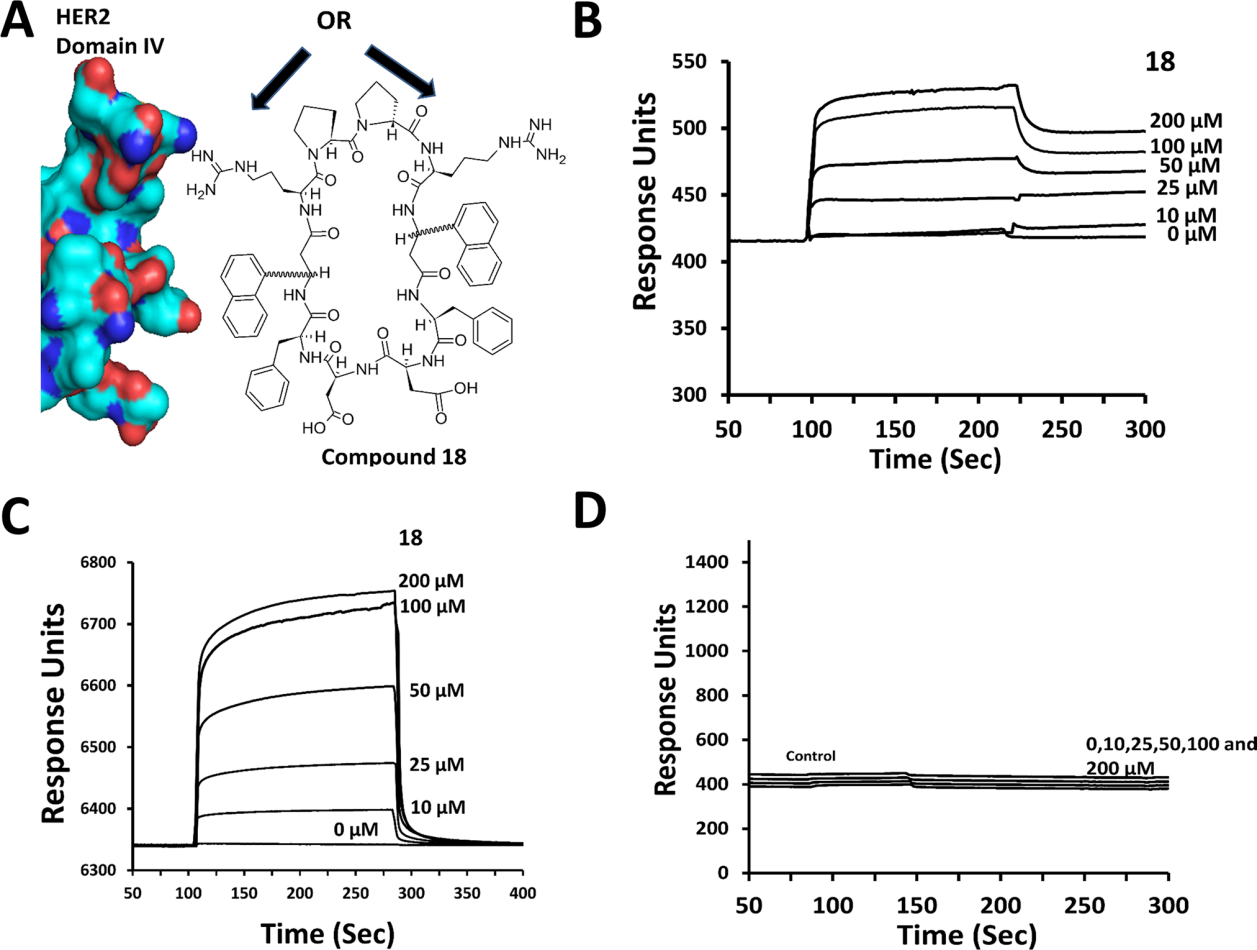
Design and binding of compound 18 to HER2 protein **(A)** Design concept of compound 18. With its double-edged sword structure, 18 can interact with domain IV of HER2 using the pharmacophore on either of its surfaces (arrows) and inhibit PPI of HER2 with other EGFR. (**B**) Binding of compound 18 to HER2 ECD (domains I to IV) analyzed by SPR. HER2 ECD was immobilized and compound 18 was used as analyte. Sensorgram shows the association and dissociation phases of compound 18 binding to HER2 ECD. (**C**) Binding of compound 18 to domain IV of HER2 at various concentrations. (**D**) Control compound at various concentrations. There was no change in the response of the sensorgram compared to control with protein surface, suggesting the absence of binding by control compound.

Currently monoclonal antibodies trastuzumab and pertuzumab that target the HER2 extracellular domain are being tested/used for treating breast cancer [14, 15]. Trastuzumab is known to bind to domain IV while pertuzumab binds to domain II of the extracellular region of HER2 [16–19]. The mechanism of action of trastuzumab is not completely deciphered; different mechanisms such as diminishing receptor signaling, antibody-dependent cellular toxicity (ADCC), and inhibition of extracellular domain cleavage have been proposed [20]. In contrast, pertuzumab is known to act by inhibiting the dimerization of EGFR [19]. Antibodies have limitations in terms of shelf-life, delivery, and immunogenicity [21]. Our aim was to design peptidomimetic dimerization inhibitors that would inhibit the heterodimerization of EGFR by hindering their protein-protein interaction sites. We designed a novel symmetric cyclic peptidomimetic compound 18 (Figure 1) [22] that inhibits HER2:HER3 and EGFR:HER2 heterodimerization. With the L-Pro-D-Pro sequence in the cyclic structure, compound 18 exhibits a β-turn/β-hairpin structure in solution. Antiproliferative activity of this compound in various cancer cell lines indicated that compound 18 exhibits specificity towards HER2-overexpressed cancer cell lines with an IC_50_ value in the low nanomolar range. When the chirality of β -amino acids in the peptide was changed, the activity of the compound relatively decreased. We also investigated L-Pro-D-Pro chirality, and our results indicate that the biological activity of compound 18 depended on the chirality of the Pro-Pro sequence as well as the β-amino acids in the peptidomimetic. Thus, a chiral switch in the compound affects its binding to HER2 and thereby its activity. The most active chiral compound 18 was evaluated for its ability to suppress tumor growth in a xenograft model of breast cancer. Compound 18 was able to suppress the proliferation of breast tumor *in vivo* and was also able to inhibit HER2:HER3 heterodimerization. Furthermore, we also evaluated the antiproliferative activity of compound 18 in EGFR mutated lung cancer cell line NCI-H1975. Compound 18 along with erlotinib, a TKI exhibited a synergistic effect on EGFR mutated lung cancer cell line. Given that compound 18 is a peptidomimetic, it has advantages compared to antibodies or peptides in terms of stability and enzymatic degradation [23]. To the best of our knowledge, no cyclic peptidomimetic highly specific for HER2 protein among EGFR, that targets domain IV of HER2, inhibits HER2:HER2 dimerization has been reported in the literature.

## RESULTS AND DISCUSSION

### Design

Our strategy was to minimize the heterodimerization of EGFRs and ultimately inhibit cell signaling for growth and survival of cancer cells. The design concept of the peptidomimetic (compound 18, Figure 1A, Table 1) that inhibits protein-protein interaction (PPI) of EGFR is unique in the sense that the designed molecule has symmetric structure with functional groups on either side of the molecule that can bind to HER2 protein and inhibit PPI of HER2 with other EGFR. In our previous studies, via different approaches we reported biological activity of peptidomimetics compounds 5 and 9 (Table 2) that inhibit PPI between EGFR-HER2 and HER2-HER3 [24–27]. However, linear peptidomimetics compounds 5 and 9 are susceptible to enzymatic degradation *in vivo*. To prevent enzymatic peptide degradation, [28, 29] strategies including backbone modification, side-chain substitution, the use of D-amino acids, cyclization, and termini modification are usually employed [30]. Since compounds 5 and 9 have functional groups that specifically bind to HER2 domain IV and inhibit PPI of EGFR, we wanted to retain the functional groups of compounds 5 and 9 and create a cyclic structure for increased *in vivo* stability.

**Table 1:** Antiproliferative activity of compound 18 in cancer cell lines

		IC_50_ (µM)				
Compound	Structure	BT-474 (+)	SKBR-3(+)	MCF-7 (–)	SKOV-3(+)	Calu-3 (+)
**18**	Cyclo(1,10)PpR (***R***) Anapa-FDDF -(***R***)-Anapa)R	0.197±0.055	0.194 ± 0.046	> 50	0.853 ± 0.102	0.018 ± 0.013
CP*	H_2_N-K(3-amino-biphenyl propionic acid)F-OH	> 100	> 100	> 100	> 100	> 100
	Lapatinib		0.013 ± 0.003			

Small letters in a sequence refer to D–amino acids and capital letters refer to L-amino acids. Anapa is 3-amino-3-(1-napthyl propionic acid). BT-474 and SKBR-3 are HER2-overexpressing breast cancer cells. MCF-7 are breast cancer cells that do not overexpress HER2. SKOV-3 and Calu-3 are HER2-overexpressing ovarian and lung cancer cells, respectively. (+) and (–) indicate HER2-positive and HER2-negative, respectively.

*CP refers to control peptide; in MCF10-A, a non-cancerous breast cell line IC_50_ of compound 18 was 40 µM.

**Table 2:** Antiproliferative activities of compound 18 and analogs in SKBR-3 cell lines along with docking energy of the lowest energy docked structure

Code	Sequence	Antiproliferative activity (IC_50_ µM) SKBR-3 cells	Docking energy kcal/mol	Docking Position on HER2 domain IV*
**18**	Cyclo(1,10)P^1^p^2^R^3^(***R***-Anapa)^4^F^5^D^6^D^7^F^8^(***R***-Anapa)^9^R^10^	0.194 ± 0.046	–10.75	PPI site
**18-1**	Cyclo(1,10)P^1^p^2^R^3^(***S***-Anapa)^4^F^5^D^6^D^7^F^8^(***R***-Anapa)^9^R^10^	> 50	–4.78	Above PPI site
**18-2**	Cyclo(1,10)P^1^p^2^R^3^(***R***-Anapa)^4^F^5^D^6^D^7^F^8^(***S***-Anapa)^9^R^10^	0.743 ± 0.35	–5.0	PPI site
**18-3**	Cyclo(1,10)P^1^p^2^R^3^(***S***-Anapa)^4^F^5^D^6^D^7^F^8^(***S***-Anapa)^9^R^10^	41.08 ± 3.2	–5.22	Above PPI site
**18-4**	Cyclo(1,10)p^1^P^2^R^3^(***S***-Anapa)^4^F^5^D^6^D^7^F^8^(***S***-Anapa)^9^R^10^	0.456 ± 0.13	–10.39	Above PPI site
**18-5**	Cyclo(1,10)p^1^P^2^R^3^(***R***-Anapa)^4^F^5^D^6^D^7^F^8^(***R***-Anapa)^9^R^10^	12.77 ± 1.9	–9.63	Above PPI site
**5**	NH_2_-Arg-Anapa-Phe-OH*	0.396 ± 0.022	–12.06	
**9**	NH_2_-Arg-Anapa-Phe-Asp-OH*	0.445 ± 0.032	–5.48	
**20**	Cyclo(1,10)GPR- (Anapa)FDEFWR*	> 100	–5.83	
**21**	Ac-f(Anapa)r-NH_2_*	0.373 ± 0.150	–6.2	

Anapa is 3-amino-3-(1-napthyl propionic acid), *reported earlier in our studies [24–27, 61].

Capital letters refer to L-amino acids and small letters refer to D-amino acids in the peptidomimetic sequence. *Supporting information provides figure for visualization of the structures.

Based on our structure-activity relationship studies of compound 5 and analogs, [24–27, 31] we designed a cyclic compound (Figure 1A), by introducing identical functional groups on either sides of the peptidomimetic, thereby anticipating to increase the affinity of binding of the peptidomimetic to HER2 protein. The amino acid/modified amino acid sequence from parent compounds 5 and 9, Arg-Anapa-Phe-Asp, was repeated to keep the pharmacophore groups in the design, and the repeated sequence was linked by a conformational constraint Pro-Pro sequence. The peptide was cyclized by main chain cyclization for stability against enzymatic degradation. The structure thus will expose the side chains of Arg-Anapa-Phe-Asp to the receptor for binding (Figure 1A). We named the resultant structure as compound 18. To investigate the chirality of the β-amino acid necessary for activity, we synthesized *R* and *S* anapa analogs of compound 18, resulting in compounds 18–1 to 18–3. The Pro-Pro sequence is known to be important in turns in proteins and peptides. Based on the conformational analysis of peptides having a Pro-Pro sequence, it is evident that D-Pro-L-Pro sequence forms left-hand turns and L-Pro-D-Pro forms right-hand turns [32]. To investigate the effect of the Pro-Pro sequence [33] on the biological activity of the compound and further the effect of Pro-Pro and the chirality of β-amino acid anapa, we synthesized compounds 18–4 and 18–5. Table 2 provides sequences of different compounds designed, the chirality of the Pro-Pro as well as the β-amino acid.

### Antiproliferative activity of compound 18 and its anapa analogs

Evaluation of the antiproliferative activity of compound 18 in HER2-overexpressing cancer cell lines indicated that compound 18 exhibited an IC_50_ value of 194 nM in breast cancer cell lines SKBR-3 and 18 nM in HER2-overexpressing lung cancer Calu-3 cell lines. On the other hand, in MCF-7 cell lines that do not overexpress the HER2 protein, the IC_50_ was > 50 µM. In normal breast epithelial cell line MCF-10A, compound 18 exhibited antiproliferative activity with an IC_50_ value of 40 µM, nearly 200 times less than its activity against the SKBR-3 cancer cell line and nearly 2000 times less compared to a lung cancer cell line (Calu-3). We also evaluated the effect of chirality of β-amino acid and the Pro-Pro sequence in compound 18 on the antiproliferative activity. Based on the IC_50_ value of compounds the following observations were made in HER2-overexpressing cancer cell lines SKBR-3 : (a) when the Pro-Pro sequence in the molecule has L-Pro-D-Pro, the compound with (R) anapa exhibited potent antiproliferative activity with IC_50_ in the nanomolar range; (b) when D-Pro-L-Pro was introduced, the compound with (S) anapa exhibited activity in the nanomolar range (Table 2). Other analogs of compound 18 with the L-Pro-D-Pro sequence and (R) anapa and (S) anapa or (S) anapa and (R) anapa had only a moderate antiproliferative activity with an IC_50_ > 5 µM. These results indicate that the compound 18 has a chiral switch. Amongst all, compound 18 was the most effective compound that has high specificity towards HER2 overexpressing cancer cell lines SKBR-3, BT-474 and Calu-3. In order to further understand the molecular mechanism and dimerization inhibition of compound 18, we carried out *in vitro* and *in vivo* studies as described below.

### Compound 18 binds to HER2 extracellular domain

Compound 18 was designed to bind to the HER2 protein extracellular domain (ECD) and inhibit dimerization of EGFR. To show that compound 18 binds to the HER2 protein, we performed competitive binding studies with FITC-labeled compound 5, which is known to bind to HER2 ECD [31]. Varying amounts of compound 18 (0.1 to 50 µM) was incubated with HER2 expressing BT-474 cells lines along with fixed amount of FITC-compound 5 (50 µM). As the concentration of 18 increased, we observed a decrease in the fluorescence intensity of cells (Supplementary Figure 1), suggesting that compound 18 competitively binds to HER2 protein on the cell surface and possibly at the same site on HER2 protein as that of compound 5.

To confirm that compound 18 binds to the ECD of HER2, surface plasmon resonance (SPR) analysis was carried out using pure proteins of HER2 ECD (domains I to IV) and only domain IV. Protein HER2 ECD and domain IV of ECD were immobilized on CM5 chips, and compound 18 was used as an analyte at different concentrations. SPR sensorgrams showed concentration-dependent binding of compound 18 to HER2 ECD as well as to domain IV (Figure 1B and 1C). Kinetic parameters were obtained by curve fitting the 1:1 binding Langmuir equation. *k*_on_ obtained was 4.76 × 10^7^ M^-1^ s^-1^ and *k*_off_ 5.97/s and, from these values, the calculated value of K_d_ was 0.125 µM. The K_d_ value obtained was consistent with the IC_50_ of compound 18 in SKBR-3 cell lines (0.194 µM). When a control compound (Table 1) was used as an analyte, no SPR response was observed (Figure 1D), suggesting that compound 18 specifically binds to HER2 ECD and in particular to domain IV. As a control for HER2 ECD, the antibody pertuzumab, which is known to bind to domain IV was used [19]. As expected, pertuzumab displayed binding to HER2 ECD (Supplementary Figure 2). Furthermore, SPR analysis of compound 18 for binding to EGFR, HER3 and HER4 did not indicate any significant change in response, suggesting that compound 18 binds specifically to HER2 protein (data not shown).

### Compound 18 inhibits the phosphorylation of HER2 kinase

**Figure 2:**
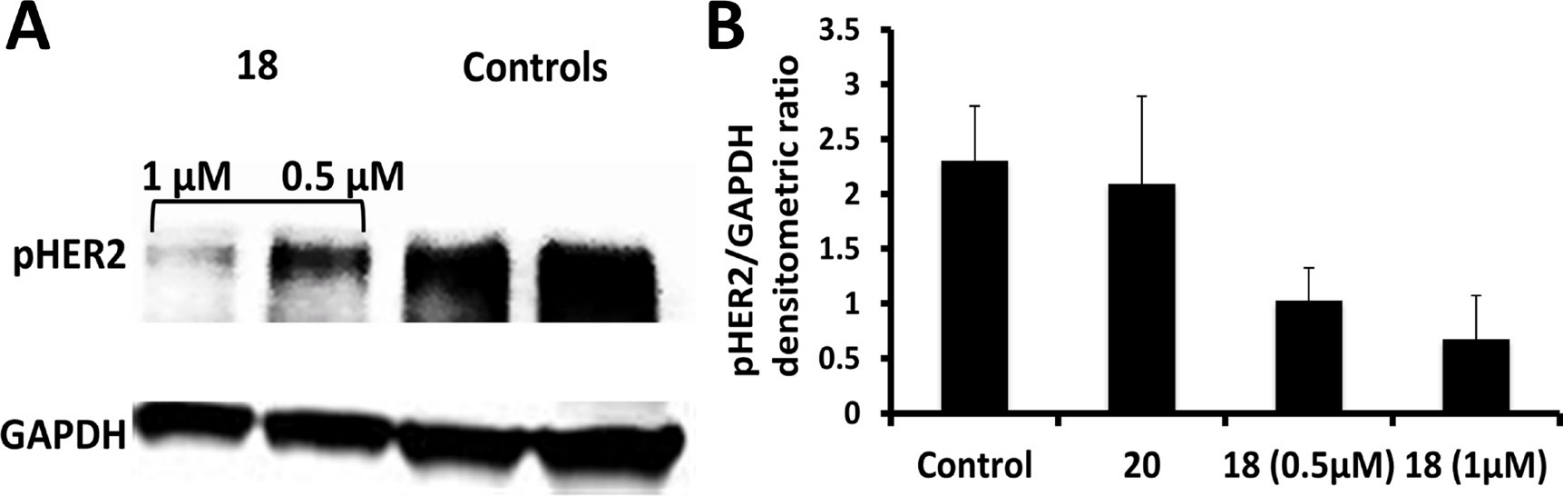
Western blot analysis of phosphorylated HER2 **(A)** BT-474 cells that overexpress HER2 protein upon treatment with compounds 18 (0.5 and 1 µM) and control without any compounds and with compound 20. (**B**) Quantification of phosphorylated HER2 from western blot compared to the controls. *P* < 0.05 for compound 18 at 0.5 and 1 µM compared to the controls.

As discussed above compound 18 binds to the ECD, inhibits the PPI of EGFR and thereby inhibiting the phosphorylation of the kinase domain. Quantitative analysis of Western blot of BT-474 and Calu-3 cells treated with compound 18 suggested that it decreases the phosphorylation of HER2 in a concentration-dependent manner (Figures 2A, 2B and Supplementary Figure 3). For Calu-3 cells, compounds 5 and 9 that are known to inhibit the phosphorylation of HER2, as well as a control compound that do not exhibit any antiproliferative activity were also used as controls. The results clearly indicate that binding of compound 18 to the ECD inhibits the phosphorylation of the intracellular kinase domain of HER2.

### Compound 18 inhibits EGFR:HER2 and HER2:HER3 heterodimerization

**Figure 3:**
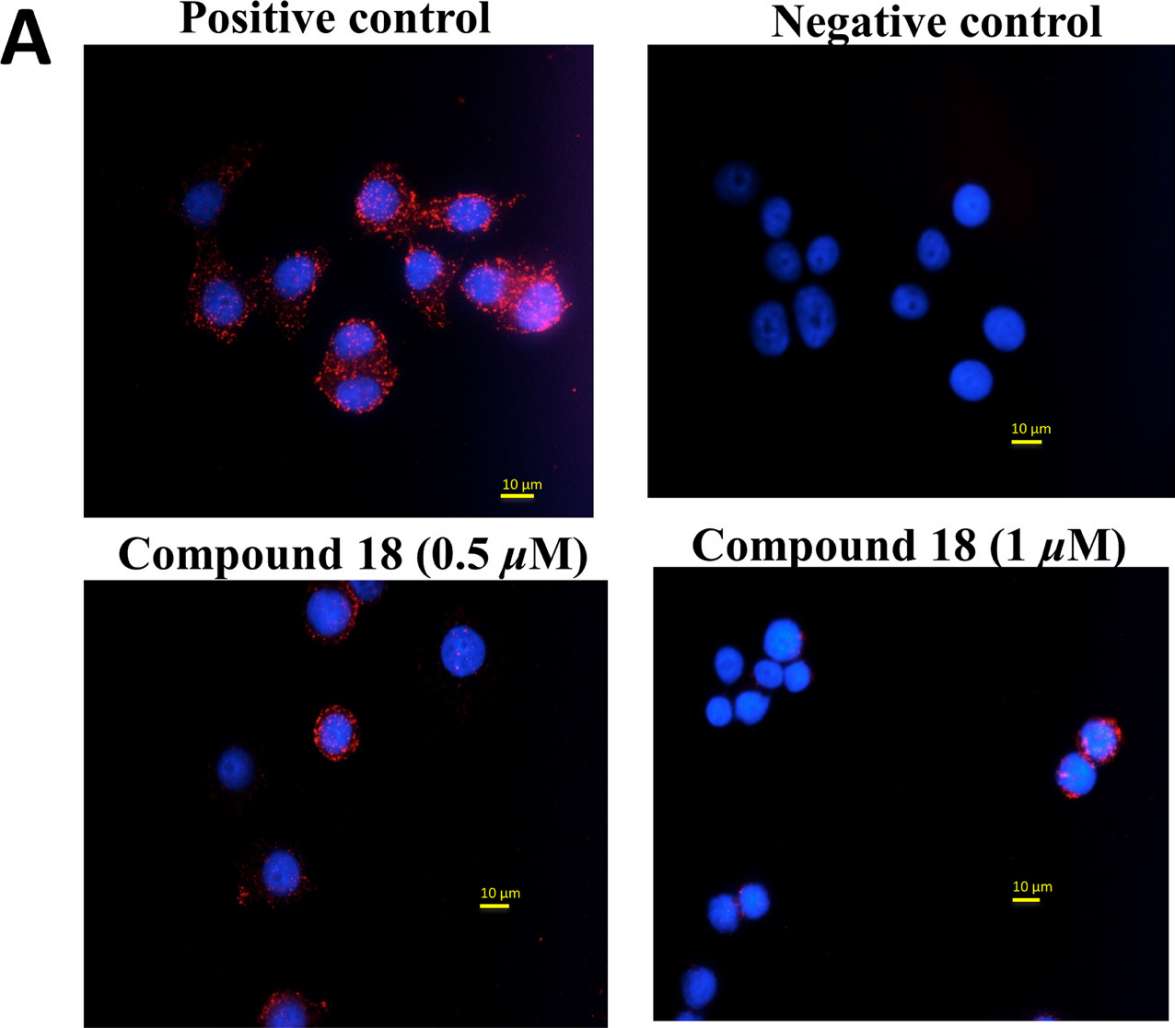

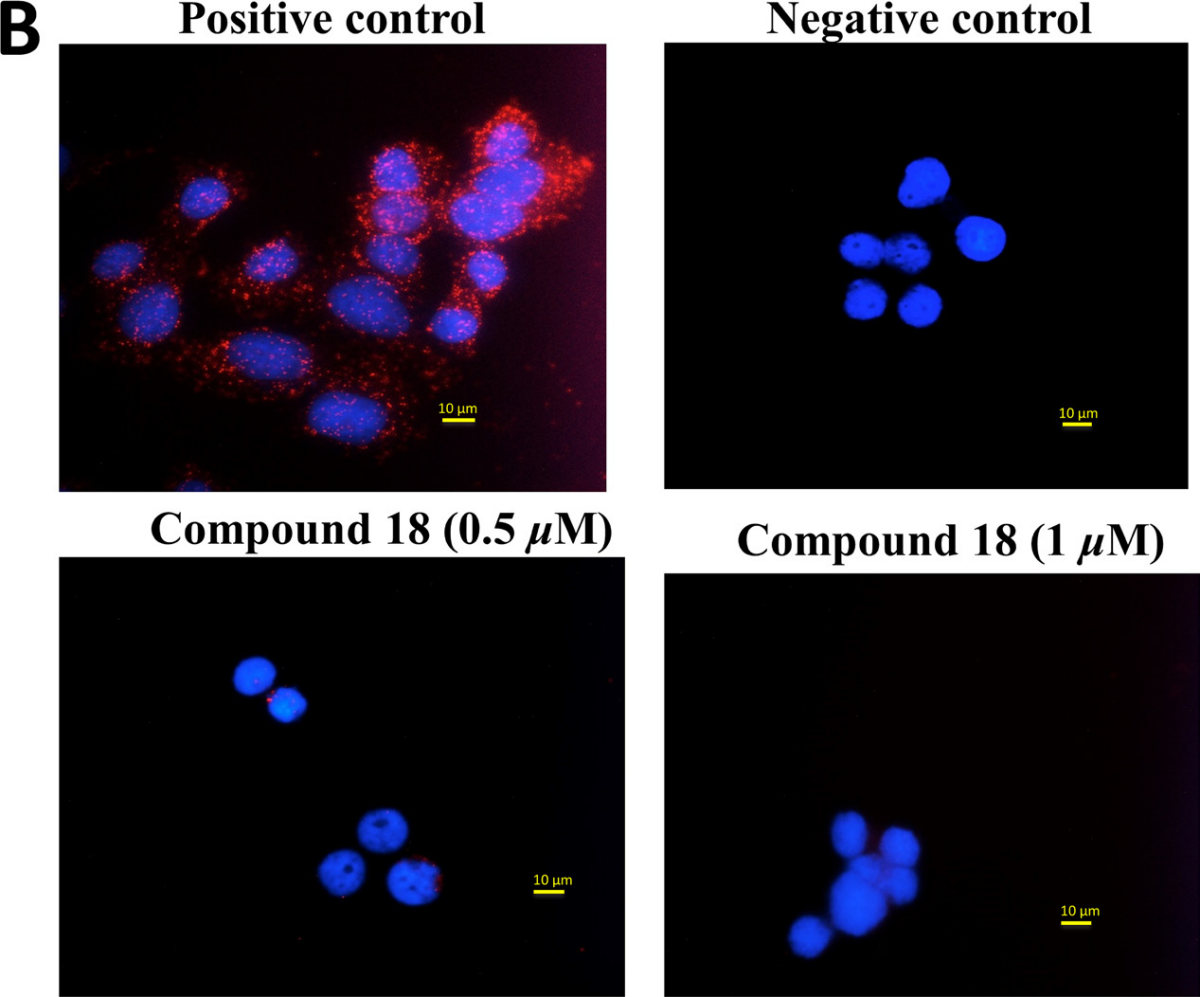
Inhibition of heterodimerization of EGFR in SKBR-3 cells determined by PLA assay (**A**) SKBR-3 cells with PLA antibodies, indicating HER2:HER3 heterodimer. Each red dot represents a heterodimer. Cells in the presence of compound 18 (0.5 and 1 µM). Note the decrease in the number of red dots, suggesting inhibition of dimerization. Nucleus was stained with DAPI. (**B**) SKBR-3 cells with PLA antibodies, indicating EGFR:HER2 heterodimer. Each red dot represents a heterodimer. Cells in the presence of compound 18 (0.5 and 1 µM). Note the decrease in the number of red dots, suggesting inhibition of dimerization (magnification 60X).

Our idea is to inhibit PPI of EGFR and, thus, modulate the cell signaling for cell growth. Compound 18 was designed to inhibit EGFR heterodimers. In order to demonstrate that compound 18 inhibits EGFR heterodimerization, we carried out proximity ligation assay (PLA) [34] and enzyme fragment complementation assay (also known as PathHunter^®^ assay) [35]. SKBR-3 cells were incubated with and without the compound, and PLA assay was carried out using antibodies for HER2:HER3 pair as well as for HER2:EGFR pair. In the absence of the compound 18, we observed a number of red fluorescence spots in the cells, indicating HER2:HER3 dimerization in SKBR-3 cells. Each red spot in the image corresponds to an HER2:HER3 heterodimer. In the presence of compound 18 at 0.5 µM and 1 µM the number of red fluorescence spots was significantly different than in the controls (Figures 3A and 3B). Our results indicate that compound 18 inhibits heterodimerization in a concentration-dependent manner (Supplementary Figure 4). We obtained similar results when we probed for HER2:EGFR dimers with PLA probes in SKBR-3 cells. These results suggest that compound 18 is a dual inhibitor of EGFR heterodimers, inhibits both EGFR:HER2 and HER2:HER3 dimerization.

**Figure 4:**
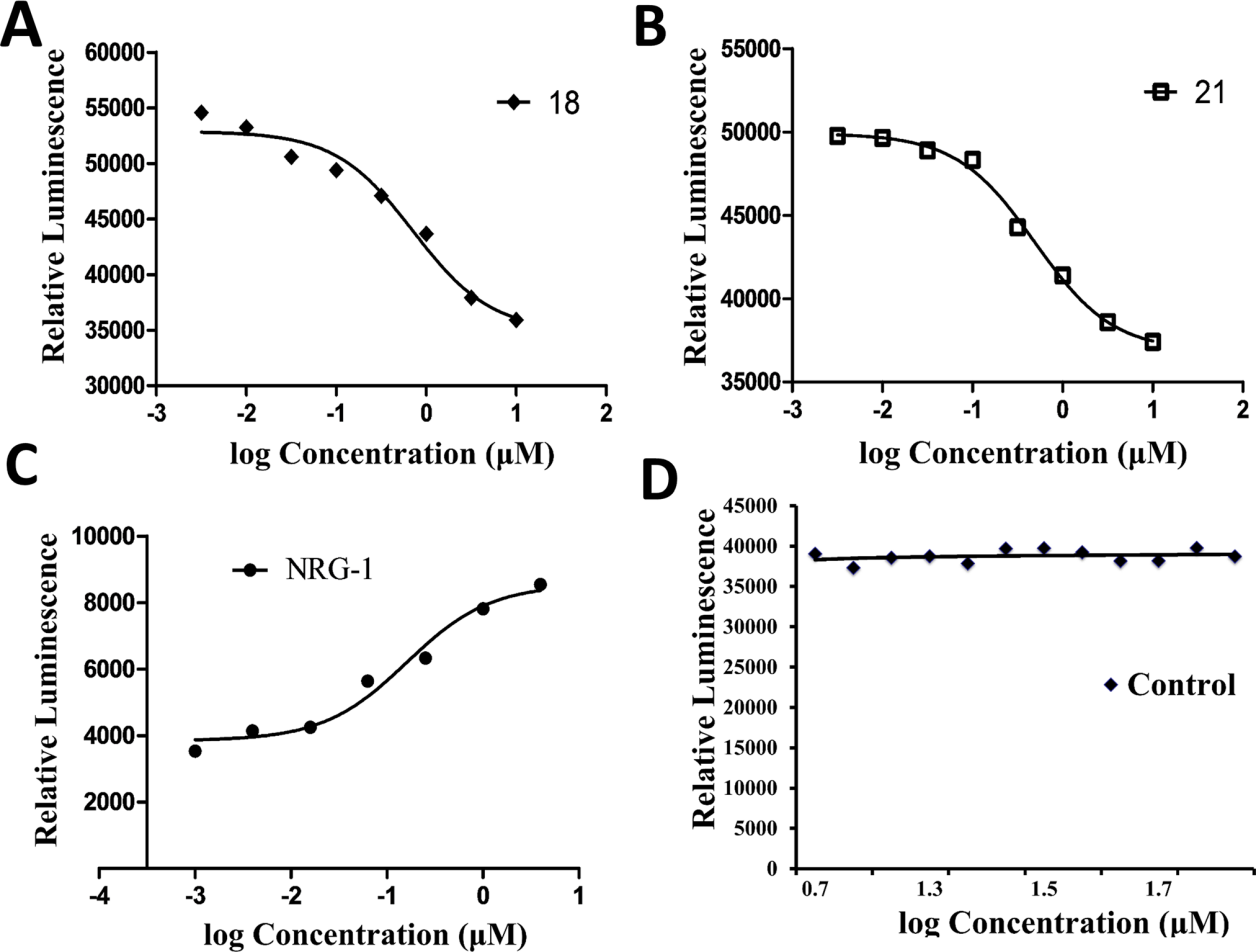
Inhibition of heterodimerization of HER2-HER3 in HER2-HER3-transfected U2OS cells by compound 18 at different concentrations using PathHunter^®^ assay **(A)** Dose-response curve for inhibition of heterodimerization by compound 18 in the presence of 0.3 µM NRG1 and (**B**) for compound 21. (**C**) Dose-response curve for agonist NRG1 and induction of dimerization (**D**) For comparison, a control compound that does not inhibit dimerization is shown. NRG1, neuregulin-1.

Inhibition of heterodimerization by compound 18 was further confirmed by enzyme fragment complementation assay. In this assay, HER2:HER3 proteins were expressed in the U2OS cell line. These EGFR were tagged with part of the β-galactosidase enzyme. Another part of β-galactosidase is linked to a SH2 domain that binds to the kinase domain of EGFR. Dimerization of HER2:HER3 in these cells can be induced by neuregulin-1, a ligand for HER3. Upon dimerization of HER2:HER3, the kinase domain is phosphorylated, and the SH2 domain binds to the kinase domain, leading to the formation of active β-galactosidase. The activity of β-galactosidase is detected by luminescence. U2OS cell lines that were transfected with HER2:HER3 were incubated with different amounts of compound 18, and β-galactosidase activity was monitored by luminescence. Figure 4A indicates that in the presence of neuregulin and compound 18 dimerization of HER2:HER3 is inhibited in a concentration-dependent manner suggesting that compound 18 inhibits HER2:HER3 dimerization and hence modulates the SH2 domain binding to TK receptor. A compound (21) an analog of 9 was also shown to inhibit HER2:HER3 dimerization (Figure 4B). A concentration dependence of induction of dimerization is observed in the case of the addition of neuregulin-1 (Figure 4C). A control compound that was also used in similar conditions did not inhibit HER2:HER3 dimerization (Figure 4D). As a positive control, lapatinib was used (Supplementary Figure 5).

### Compound 18 induces apoptosis in HER2-positive cells

Our next aim was to investigate if compound 18 induces apoptosis, we have carried out the TUNEL assay. Compound 18 at 5 and 10 µM concentrations showed positive staining for apoptosis in SKBR-3 cells compared to control suggesting that compound 18 induces apoptosis. DNAse-treated cell samples were used as positive control (Supplementary Figure 6).

### Compound 18 exhibits antiproliferative activity in EGFR mutated cell lines and has synergistic effect with erlotinib

**Figure 5:**
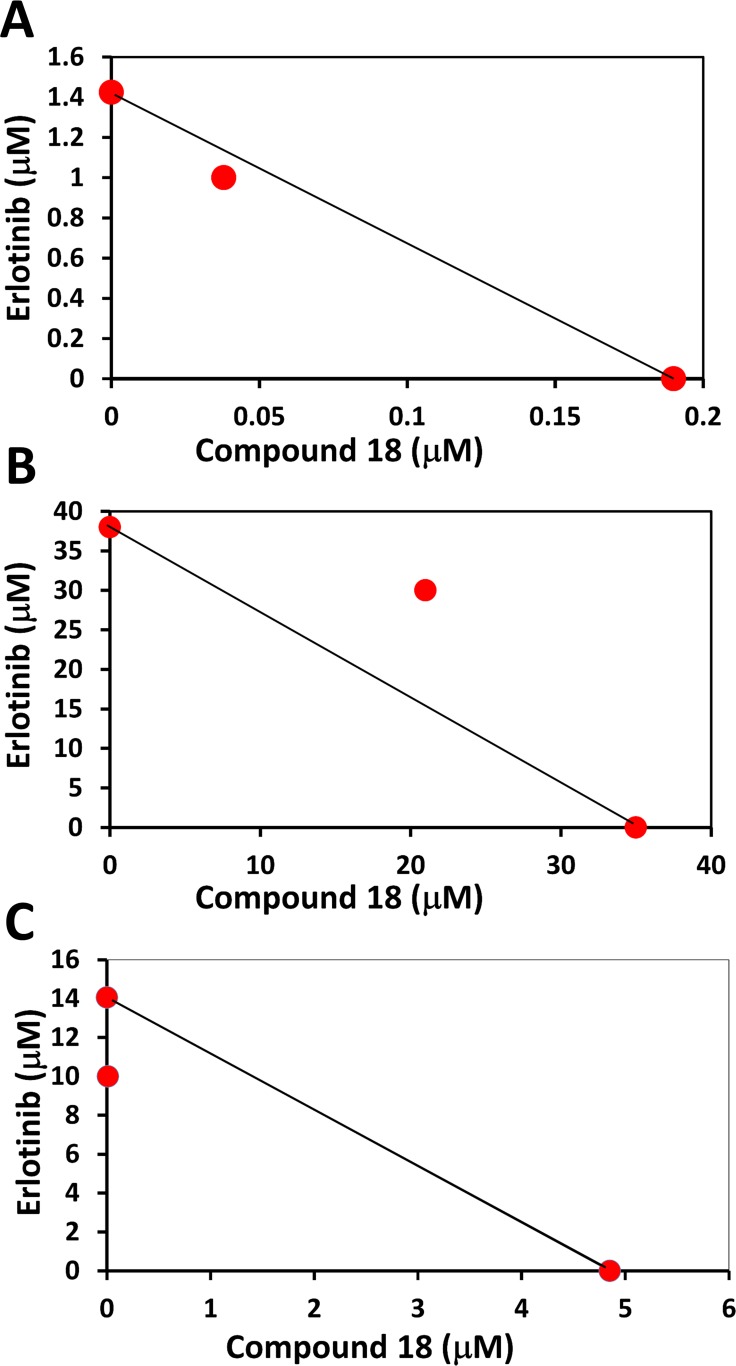
Isobologram plots for analysis of synergistic effect of compound 18 with erlotinib in different cells lines (**A**) BT-474 (HER2 positive), (**B**) MCF-7 (HER2 negative) and (**C**) EGFR mutated lung cancer cell line NCI-H1975. In BT-474 and mutated cell lines synergistic effect was seen.

Use of first generation kinase inhibitors such as erlotinib and gefitinib lead to development of drug resistance and in nearly 50% of NSCLC cases the resistance to TKI therapy is due to the presence of T790M mutation [36]. We wanted to evaluate whether compound 18 has any effect on the T790M mutation and has any synergistic effect with erlotinib on different cancer cell lines including NCI-H1975 cell lines. NCI-H1975 is a cell line that harbors the EGFR L858R/T790M double mutation [37]. Antiproliferative activity of compound 18 in NCI-H1975 was 4.85 µM which is much higher (low potency) than in HER2 overexpressing cancer cell lines, while IC_50_ for erlotinib was 14 µM in NCI-H1975 cell lines. Synergistic effect of compound 18 and erlotinib was evaluated in HER2 overexpressing BT-474, and in MCF-7 which does not overexpress HER2 as well as on NCI-H1975 cell lines. Isobolograms were plotted to determine synergistic effect [38]. Our data clearly demonstrates that compound 18 and erlotinib exhibited a synergistic effect in BT-474 and EGFR mutated lung cancer cell lines NCI-H1975 (Figure 5), whereas in MCF-7 cell lines there was no synergistic effect. These studies clearly suggest that compound 18 has a potential to be used as a therapeutic agent in lung cancer cell lines along with TKIs.

### Compound 18 delays the progression of breast cancer tumors

**Figure 6:**
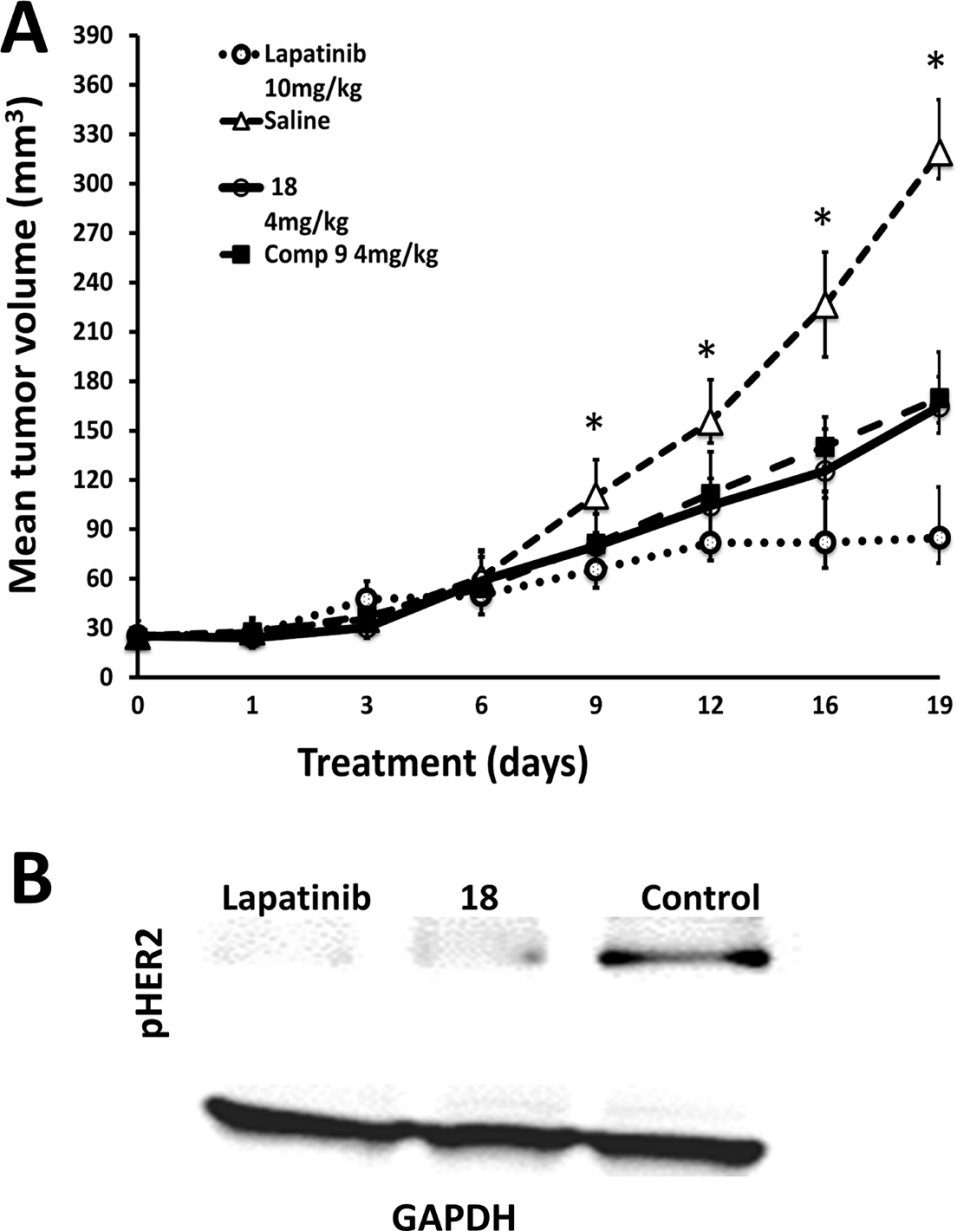
Anti-tumorigenic activity of compound 18 **(A)** Compound 18 (4 mg/kg) (thick line) delayed tumor growth in athymic nude mice significantly compared to the control group without any treatment (triangles). For comparison, compound 9 (filled squares) and lapatinib (open circles) are also shown (with standard errors). Statistical analysis indicated that for days 9–19 there was a significant difference between control and compound 18 (*p < 0.05). (**B**) Western blot analysis of tumor sections of controls and mice treated with compound 18. Tumor sections were homogenized and protein was extracted and subjected to western blot. Compound 18 showed a decrease in phosphorylation of HER2 kinase.

Our premise was, inhibition of dimerization of ECD of EGFR leads to inhibition of signaling for cell growth in tumors positive for HER2. As a proof of concept we evaluated the ability of compound 18 to inhibit the breast tumor growth in a xenograft model of breast cancer. For comparison purposes, we have used compound 9 a linear analog of compound 18 and lapatinib a kinase inhibitor [39]. Breast tumors were induced in athymic nude mice (Foxn1nu/Foxn1+, female, 4–5 weeks) by injecting BT-474 cells. Once the tumor size reached 3 mm diameter, treatment was started by injecting the compounds 18, and 9 at 4 mg/kg twice a week via intratumor injection just below the breasts. Lapatinib and PBS were used as controls. As seen in Figure 6A the tumor size in the mice of the control group with vehicle treatment increased during the course of the experiment reaching a diameter of 10 mm in 19 days. There was a delay in tumor growth and tumor size in compound 18 treated mice group. A Mann-Whitney *U* test was performed and the delay of tumor growth between vehicle treated group and compound 18 treated group from days 9 to 19 was statistically significant (*p* < 0.05). Positive control lapatinib showed a significant delay in tumor growth as expected. The delay of tumor growth between compound 18 and compound 9 was not statistically significant. To evaluate if compound 18 inhibits the phosphorylation of HER2 kinase, we carried out Western blot analysis. Results of the western blot indicated that there was a decrease in HER2 phosphorylation in tumor samples that were treated with compound 18 compared to the control (Figure 6B). To evaluate whether compound 18 inhibited the heterodimerization *in vivo,* tumor sections of compound 18 and controls were treated with primary and secondary antibodies of HER2 and HER3 and PLA assay was carried out. Slides from PLA were viewed using a fluorescence microscope for detection of HER2:HER3 dimerization. Untreated tumor sections of mice exhibited red fluorescence, suggesting heterodimerization of proteins (Supplementary Figure 7), while tumors treated with compound 18 exhibited diminished red fluorescence signals indicating a decrease in the heterodimerization of HER2:HER3 *in vivo*.

**Figure 7:**
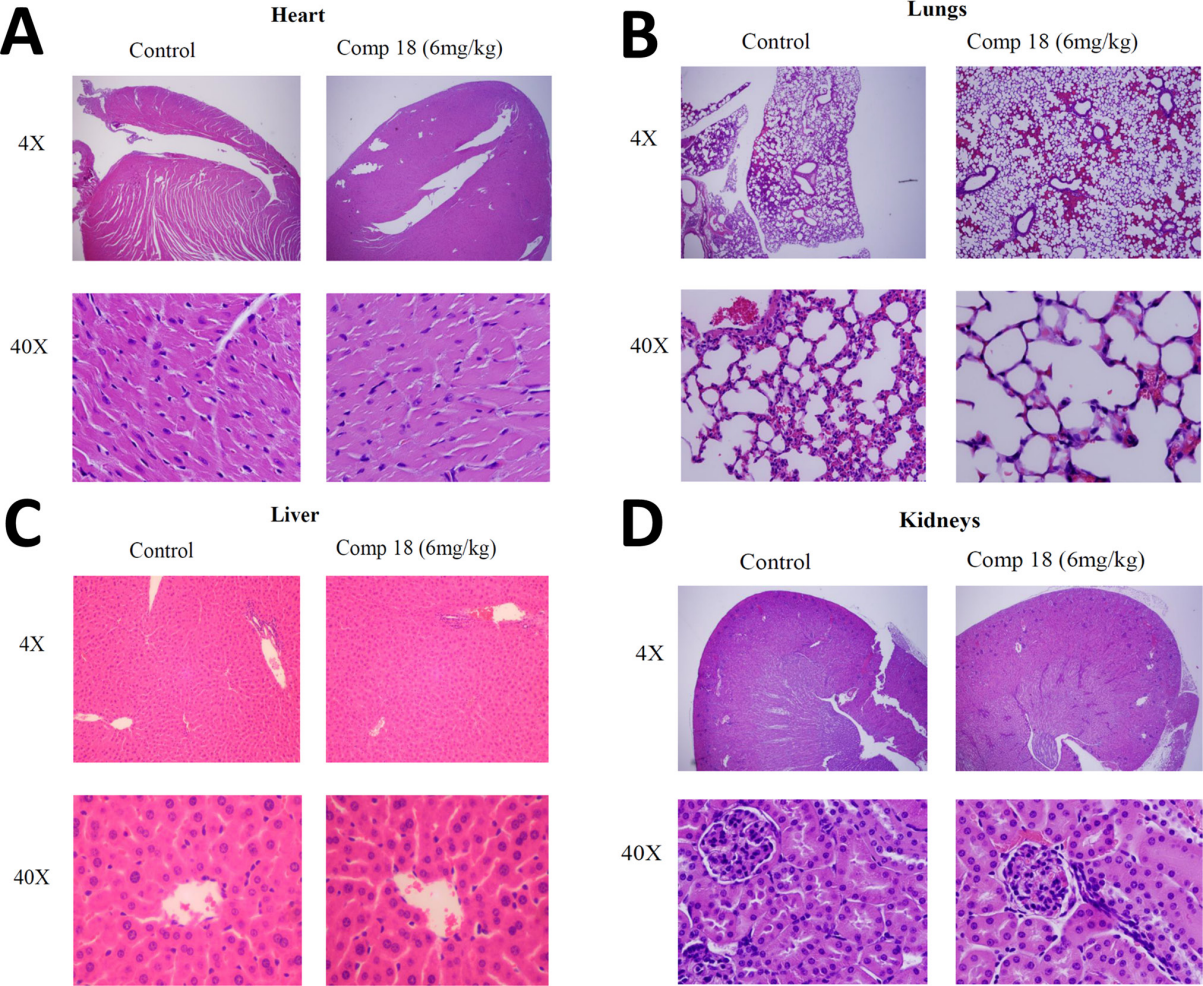
Histology studies compared organs from mice treated with compound 18 to organs from untreated control mice Compound 18 in saline (6 mg/kg) or vehicle was administered to mice i.v. Twenty-four hours after injection the mice were euthanized and organs were harvested, fixed, and prepared on slides. H&E staining of (**A**) heart, (**B**) lungs (**C**) liver, and (**D**) kidneys showed histologically normal sections without necrosis and without infiltration of inflammatory cells such as macrophages. These observations suggest that compound 18 is not toxic to the organs at the therapeutic dose used in these experiments.

We also evaluated the toxicity effect of compound 18 at therapeutic dose in mice (a dose of 6 mg/kg which is slightly higher dose that the one used for xenograft model of breast cancer). Organs such as heart, liver, kidneys, and lungs from the animals were harvested and used for histology studies. The organs were collected from the animals 24 h after administration of compound 18 and from control mice and fixed using formalin. H&E staining of heart, kidneys, lungs, and liver sections did not indicate necrosis or infiltration of inflammatory cells such as macrophages (Figure 7A–7D). These observations suggest that compound 18 is not toxic to the vital organs at the therapeutic dose used in these experiments (this was not a toxicity study *per se*).

### *In vitro* stability studies

**Figure 8:**
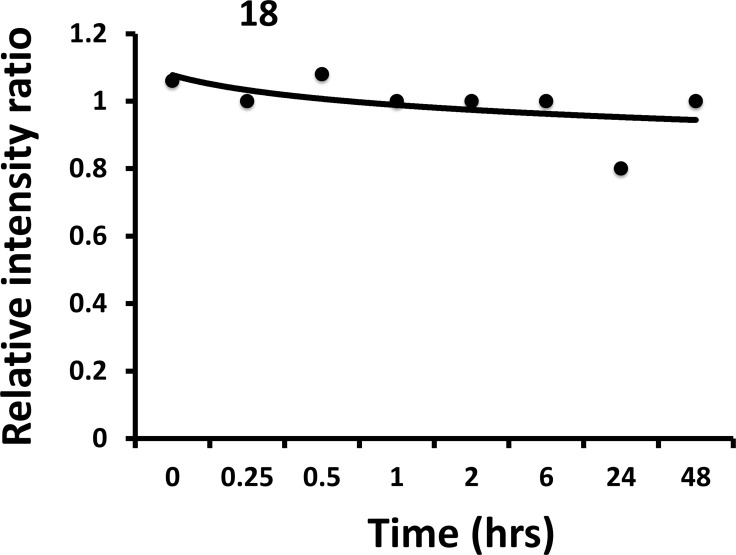
Stability of compound 18 in mouse serum (*in vitro*) Compound was incubated in mouse serum for different intervals of time and extracted. Analysis of the compound was carried out using electrospray-mass spectrometry. Internal standard was used and the ratio of intensity of the peak of compound and internal standard was used to plot the graph. The graphs indicate that compound 18 is stable even after 48 hours in mouse serum.

In our previous reports [26, 27], we described the structure and *in vivo* activity of linear analogs (compounds 5 and 9) of compound 18. Linear peptidomimetics with N- and C-termini are susceptible to enzymatic degradation *in vivo* [30, 40, 41]. The stability of cyclic peptidomimetic 18 was evaluated to verify how the cyclization will improve the stability compared to linear peptidomimetics. Mouse serum was used to evaluate the stability as the *in vivo* effect of the compound was assessed in a mouse model. Compound 20 (Table 2), a cyclic peptidomimetic was used as internal standard. Based on the time vs. relative intensity plot obtained, compound 18 was stable for 48 h (Figure 8). The half-life of compound 18 in mouse serum could not be determined because of nearly linear nature of the graph.

### NMR and molecular modeling

**Figure 9:**
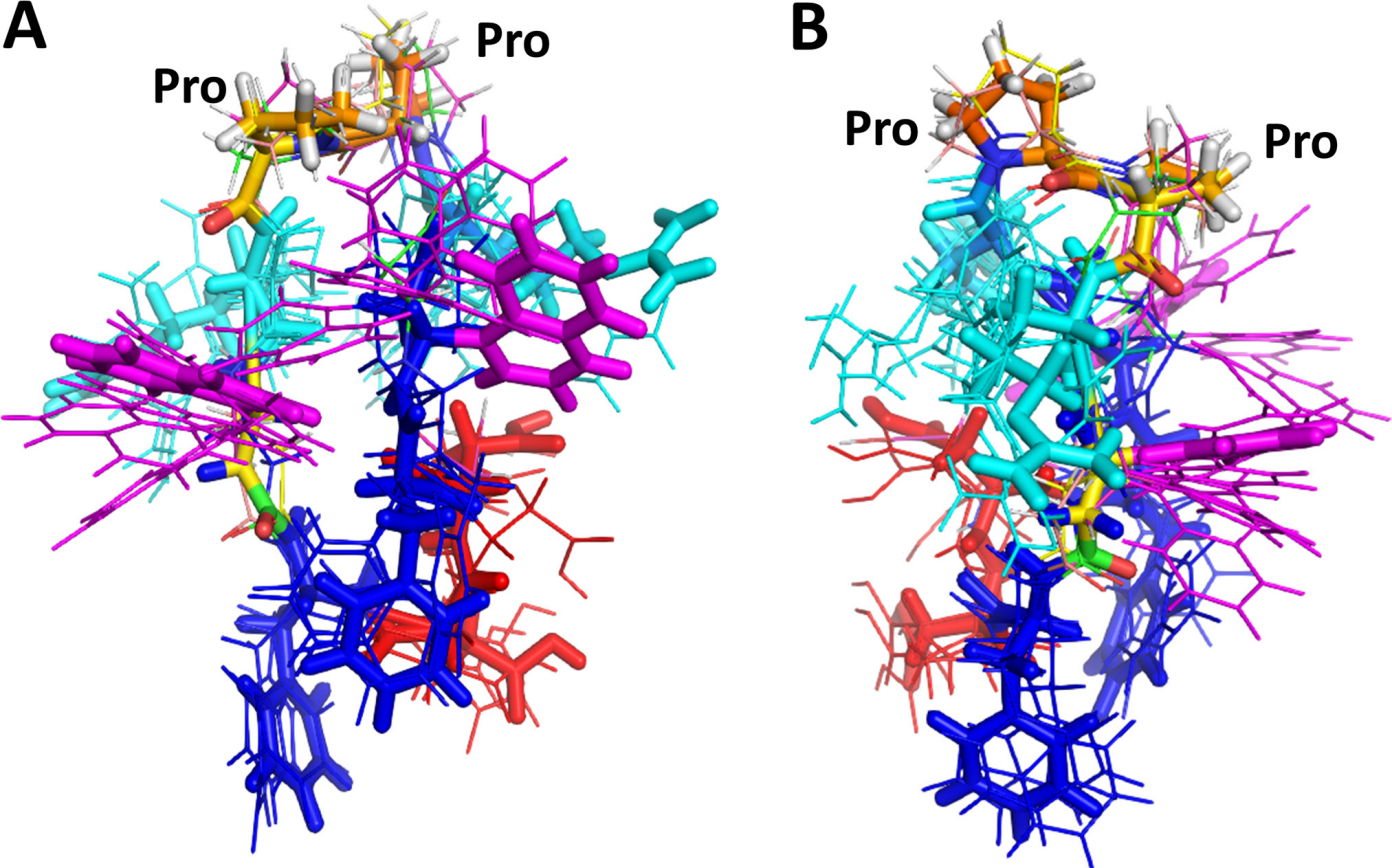
Proposed overlapped structures of compound 18 and its diastereoisomers in different orientations (**A** and **B**). Side chains of compound 18 are shown as sticks, and side chains of other diastereoisomers are shown as thin lines in different colors. Anapa-magenta, Phe-blue, Asp-red, Arg-cyan. Pro-Pro sequence in the compound is indicated as a reference. Arg and Anapa side-chain orientations were different in different isomers whereas Phe and Asp were clustered around the same region.

To explain the observed IC_50_ values of compound 18 and analogs, the effect of chirality of beta amino acid as well as Pro-Pro sequence, NMR and molecular modeling studies of these compounds were carried out. 2D TOCSY NMR pattern of compound 18 and analogs indicated that there are slight differences in the resonances for amino acids for the analogs of compound 18–3 and 18–4 compared to NMR spectra of compound 18 (Supporting information). In particular, Anapa9 Phe5 amide resonances exhibited different chemical shifts in 18, 18–3 and 18–4. In 18–4 amide resonances were not well dispersed suggesting flexible nature of the compound 18–4. The structure of compound 18 was generated using NMR and simulated annealing approach. The number of NOE constraints obtained from NMR experiments were limited to compound 18 and analogs. With the limited number of NOE constraints, energy minimization and MD simulations were used to generate the possible conformations of the peptidomimetic structure in solution. Using docking studies, we propose a possible binding site for the compounds and also explain how the chiral switch in the structure of compound 18 leads to a change in activity of diastereomers. First, we aligned the diastereomers of compound 18 (Table 2) using molecular modeling software and observed the orientation of different functional groups. As can be seen in Figures 9A and 9B there is a notable difference in the orientation of *β*-naphthyl functionalities due to the change in stereochemistry. When these structures were subjected to docking with HER2, we observed that all the isomers interact mainly with a hydrophobic binding pocket. The polar interactions in most of the structures such as electrostatic interactions between side chains of Arg in compound 18 and Asp570, Gly581, Glu544, Val582 of HER2 protein remained the same. However, there was a difference in hydrophobic interactions of *β*-naphthyl and Phe side chain in the structure of ligand to the protein. In compounds 18 and 18–4 that represent the active diastereomers, both the *β*-naphthyl groups in the structure aligned toward the hydrophobic pocket and interact with Pro543, Phe573, Pro584, and Pro590 of HER2. These important hydrophobic interactions of *β*-naphthyl were observed in docking of 18 and 18–4 isomers. Docking results of 18 and 18–4 indicate that one of the *β*-naphthyl groups interacts with HER2 while the other is oriented away from the hydrophobic binding pocket. Although the loss in activity of these isomers can be explained from the free energies of docking calculated (Table 2), the other inactive isomers showed favorable docking energies (18–1, 18–2 and 18–5) in the range of –4.78 to –9.63 kcal/mol. This can be attributed to the interactions with other residues that seem to be unimportant for PPI, as they are away from the PPI surface. To explain this, we compared the PPI binding interface of EGFR homodimer and compared with HER2 in the similar region. For compounds 18 and 18–2, the lowest energy docked structures were near the PPI site (Table 2) (Supplementary Figure 8), whereas compounds 18–1, 18–4 and 18–5 were docked above the PPI site. This clearly explains that although compounds 18–4 and 18–5 docked with lowest docking energy comparable to that of compound 18, the antiproliferative activity was not comparable to compound 18 because 18–4 and 18–5 were not blocking the PPI of HER2 with HER3 and EGFR. Compound 18–2 bound to the same site as compound 18; however, the docking energy was -5 kcal/mol suggesting that compound 18–2 is not as potent as compound 18. In summary, these docking studies suggest that the *β*-naphthyl group and the Phe side chain participate in key interactions between compound 18 and HER2 and that a change in chirality at anapa and Pro can result in binding of compounds to HER2 on location near PPI site but not on PPI site resulting in loss of antiproliferative activity. The peptidomimetic compound 18 we designed has a positively charged amino acid Arg, hydrophobic groups such as *β*-naphthyl, and Phe in the sequence. It is known that the PPI interface is dominated by amino acid residues such as Trp, Arg, Tyr, and Phe [42–44]. Structurally *β*-naphthyl group is an analog of amino acid Trp. With the functional groups of amino and β-amino acids, Arg, *β*-naphthyl, and Phe, compound 18 mimics the PPI interface and hence inhibits PPI of EGFR. Such peptidomimetics are not only useful as therapeutic agents but will also serve as probes to better understand the underlying mechanism of PPI. The crystal structure of a homodimer of EGFR with domain IV interactions has been reported. However, the crystal structure of homo- or heterodimers of EGFR:HER2 or HER2:HER3 have not been reported to date. Using the homodimer structure of EGFR, we overlapped domain IV of HER2 (PDB 3N85) [45] with domain IV of EGFR (PDB 3NJP) [46] to identify potential interaction sites (Tyr588, Leu586, Pro590, Trp592, Thr 609) on HER2 DIV (Supplementary Figure 9). Interestingly, using docking studies, we found that the amino acids interacting with compound 18 correspond to the residues that are important for PPI of HER2 with EGFR. Compound 18 binds to domain IV of HER2, presumably near the PPI site of heterodimers of EGFR and inhibits PPI and hence the signal for cell growth.

Compound 18 designed to inhibit PPI of both EGFR:HER2 and HER2:HER3 is a cyclic peptidomimetic that exhibits stability in serum. In a xenograft model of breast cancer, the compound 18 produced a significant reduction in tumor growth compared to the control. PLA carried out *in vitro* on HER2 overexpressing breast cancer cells as well as on tumor sections suggested that compound 18 inhibits the HER2:HER3 heterodimerization under *in vitro* and *in vivo* conditions. These studies suggest that inhibition of HER2 heterodimerization with other EGFR like compounds hinders cell signaling and leads to controlled cell proliferation and tumor growth.

In summary, based on the data presented here we can conclude that peptidomimetic compound 18 binds to ECD of HER2 specifically and inhibits ECD heterodimerization of EGFRs thereby abrogating the downstream signaling by EGFR proteins. When the chirality of beta amino acid (anapa) was changed in the peptidomimetic sequence, the antiproliferative activity of the resulting compound was changed. Furthermore, the chirality of the Pro-Pro sequence seemed to play a role in the orientation of side-chain functional groups in the peptide that could affect the biological activity. Thus, there is a chiral switch in compound 18.

## MATERIALS AND METHODS

### Material

Fmoc-protected amino acids were obtained from AAPPTEC (Louisville, KY) and EMD Biosciences (San Diego, CA). The resin was purchased from Chem-Implex (Wood Dale, IL). All cell lines studied were purchased from American Type Culture Collection (ATCC, Manassas, VA). Cell Titer-Glo^®^ reagent was purchased from Promega (Madison, WI). NMR solvents were obtained from Cambridge Isotope laboratories, Inc., Tewksbury, MA.

### Synthesis

Compound 18 and diastereoisomers were synthesized using standard solid-phase Fmoc chemistry using 2-chlorotrityl chloride resin (3 g, 0.89 mmol/g) [47, 48]. 2-Chlorotrityl chloride resin (3g, 0.89 mmol/g) was swollen in 30 mL of dichloromethane for 30 min. The resin was then loaded with a solution of Fmoc-Pro-OH (900 mg, 2.67 mmol) and DIEA (2.325 mL, 13.35 mmol) in 30 mL of dichloromethane. The resin mixture was agitated for 2 h and was then filtered and washed with NMP (30 mL, 6 × 30 s), DCM/MeOH/DIEA (80:15:5, 2 × 15 min), NMP (5 × 30 s), and dichloromethane (5 × 30 s). The resin was dried under vacuum overnight, after which the substitution level was checked by UV absorption of the Fmoc-piperidine adduct. After the substitution level had been determined, the resin was swollen with DMF (2 × 10 min) and then deprotected with 30 mL of 20% piperidine/NMP (2 × 5 min). The resin was then washed with DMF (5 × 30 s) and dichloromethane (5 × 30 s) and dried under vacuum overnight. The deprotected resin was stored under an inert atmosphere at –20 °C. Using an automatic peptide synthesizer, the remaining portion of compound 18 was synthesized. H-Pro-CTC resin (91 mg, 0.55 mmol/g, 50 µmol) was placed in a 10 mL reaction vessel, which was placed on the synthesizer. The resin was swollen on the synthesizer in DMF (1 × 30 min) as part of the first coupling cycle. 5 eq of Fmoc-protected amino acids, and 5 eq of HCTU dissolved in 2 mL of a 0.4 M solution of 4-methylmorpholine in NMP and added to the resin. Coupling and deprotection sequence was repeated for each of the remaining amino acids in the sequence. After the last amino acid had been added to the peptide, the final Fmoc-protecting group was removed. The peptide was cleaved from the resin by adding 2,2,2-trifluoroethanol:dichloromethane (2 mL, 1:1) to the resin and agitating for 3 h. The dried solid was dissolved in 100 mL of tetrahydrofuran (THF)/DMF (4:1) and 3-hydroxy-3H-1,2,3-triazolo[4,5-b]pyridinato-O)tri-1-pyrrolidinyl-phosphorus hexafluorophosphate (PyAOP) (4 eq, 104 mg) and DIEA (7 eq, 70 µL) was added and cyclization was carried out by agitating the solution for 2 h. The crude cyclic peptidomimetic formed was then treated with a deprotection cocktail (4 mL of TFA:water:TIPS, 95:2.5:2.5) for 3 h to remove the side chain protecting groups. The peptidomimetic solution precipitated with 30 mL of cold diethyl ether. Precipitate was dried and the solid was dissolved in 4 mL of 0.1% TFA/water, frozen, and lyophilized to yield a white powder as the crude peptide. The compounds were characterized by HPLC, mass spectrometry, and circular dichroism spectroscopy. Calculated mass for compound 18, 1425.5893, exact mass m/z 1425.699, > 95% purity. Analytical data for compound 18 and analogs are provided in supporting information (Supplementary Table 1 and Supplementary Figures 10–20).

### Antiproliferative activity

Cellular assays were performed using BT-474, SKBR-3, Calu-3, MCF-7, and MCF-10A cells (ATCC). Antiproliferative activity was measured by CellTiter-Glo^®^, [49] a luminescence-based cell viability assay. In a 96-well plate, 1 × 10^4^ cells/well were seeded and incubated overnight at 37°C and 5% CO_2_. Stock solutions of peptidomimetics were prepared by dissolving 1.5 mg/mL of compounds in DMSO. Stock solution of the compounds were diluted using the serum-free medium to prepare solutions of different concentrations of compounds. Compounds in the medium were added to the wells in triplicate and incubated for 72 h. As controls, cells treated with 1% sodium dodecyl sulfate and 1% dimethyl sulfoxide were used. At the end of the experiment, CellTiter-Glo^®^ reagent was added, and luminescence was measured by a plate reader. A dose response curve was generated using luminescence vs concentration of compounds and IC_50_ values were obtained using Prism^®^ (GraphPad Software, La Jolla, CA). Experiments were repeated at least three times to obtain standard deviation values. EGFR mutated lung cancer cell lines NCI-H1975 (ATCC^®^ CRL 5908™) was obtained from ATCC, and antiproliferation assays were carried our as describe above. Erlotinib was from Sigma (Sigma-Aldrich Chemie GmbH). To evaluate the synergistic effect of compound 18 with erlotinib, first a dose response curve was generated for erlotinib, and compound 18 alone in different cell lines including EGFR mutated NCI-H1975 cells. Then cells were treated with different concentrations of compound 18 (0.005–10 μM) along with fixed concentrations of erlotinib. Fixed erlotinib dose of 1 μM, 1 μM, 30 μM, and 10 μM were selected for BT-474, Calu-3, MCF-7 and mutated cell line NCI-H1975 respectively. The isobologram was generated by plotting IC_50_ values (50% cell growth inhibition) of compound 18 and erlotinib, alone and in combination. From the isobologram, synergistic effect of compound 18 with erlotinib was determined.

### Competitive binding assay

BT-474 cell lines were coated on 96 well plates and after 24 hrs cells were washed. Varying amounts of compound 18 (0.1 to 50 µM) was incubated with HER2 expressing BT-474 cells lines along with fixed amount of FITC-compound 5 (50 µM). Cells were washed after 1 hr and fluorescence was measured using a Biotek plate reader with excitation λ 485 nm and emission λ 528 nm. Readings were in triplicate. A graph of relative fluorescence with respect to concentration of compound 18 was plotted. As control fluorescence from FITC-compound 5 and cells without any treatment were used.

### Western blot

BT-474, Calu-3, and SKOV-3 cells were treated with compounds at 1 µM and lapatinib (positive control) at 0.07 µM. Cells without any treatment and cells treated with control compound were employed as negative controls in these studies. Cells were incubated for 36 h, washed and trypsinized. The cell lysate was prepared using cell lysis buffer containing protease inhibitor and phosphatase inhibitor. The protein concentration in each sample was determined using Bradford’s assay. 40 μg of protein from each sample was loaded on Novex^®^ 4–20% tris-glycine gels and western blot analysis was carried out as described in our previous publication [26]. Antibodies for the detection of total HER2 protein (t-HER2) and phosphorylated HER2 protein (p-HER2) were used at 1:3000 dilutions. After addition of the substrate and enhancer solutions from a Super Signal-enhanced chemiluminescence kit (Pierce, Rockford, IL) to the membrane, the images were captured using C-Digit Blot Scanner (LI-COR Biotechnology, Lincoln, NE). A representative Western blot image was used for the final presentation.

### Inhibition of heterodimerization by enzyme fragment complementation assay

A Pathhunter assay [35] kit was obtained from DiscoverX, and enzyme fragment complementation assay was carried out. U2OS cells transfected with HER3:HER2 were used for the assay. After cells were attached to the wells, they were incubated with compound 18 and controls at different concentration in the presence of neuregulin-1 (NRG-1) at 0.3 µM concentration. After 24 h of incubation, reagents were added, and luminescence was measured and compared with a control without any compound. A plot of luminescence versus concentration was obtained to measure the dimerization of HER3:HER2 and its inhibition by compound 18.

### Proximity ligation assay (PLA)

Proximity ligation assay was performed as described previously [34, 27]. Cells were incubated in 8-well slide plates with compound 18 and controls (no compound and pertuzumab) for 24 h and fixed using cold methanol. Fixed cells were used for PLA assay. Cells were incubated with primary antibodies for 24 h and washed, and secondary antibodies PLA+ and PLA− were added. After incubation and washing, PLA detection reagent was added, and the cells were covered with a glass plate after mounting medium was added. Slides were viewed using an inverted microscope and a multiphoton microscope. Images were obtained at 40 and 60X.

### Surface plasmon resonance analysis

Surface plasmon resonance analysis [50] was performed using GE X100. Extracellular domains of proteins HER2, EGFR, HER3, and HER4 purchased from Leinco Technologies (St. Louis, MO). Pertuzumab was generously provided by Genentech Inc. Each of these proteins was immobilized on a CM5 chip. Compound 18 and controls were used as analytes. Compound 18 at different concentrations (0 to 200 µM) was passed through an SPR chip, and binding kinetics were analyzed for each of the EGFR bindings. A blank subtracted sensorgram for compound 18 was represented.

### TUNEL assay

Ten thousand SKBR-3 cells were added to each well of Lab-Tek^®^ 8-chamber slides and incubated overnight for cell attachment. Compound 18 (10 µM) was added and incubated for 48 h at 37°C. Cells were washed twice with cold PBS. Cell fixation was achieved using 10% buffered formalin followed by permeabilization of membranes using 0.2% Triton X-100 in PBS at room temperature. 100 µL of rTdT reaction mix containing biotinylated nucleotide mix was added. The slide was placed in a humidity chamber and incubated for 60 min at 37°C. With the addition of dilute saline-sodium citrate (SSC) solution (20X provided with the kit diluted to 2X), the reaction was stopped. Three PBS washes were made to ensure the removal of remaining unincorporated biotinylated nucleotides on the slide. Treatment with 0.3% hydrogen peroxide was done to block the endogenous peroxidases in the cells. The slide was incubated for 30 min at room temperature with dilute streptavidin HRP solution (1:500 in PBS). Diaminobenzidine (DAB) was added to stain the slide. After drying, DAPI was used to counterstain the nuclei, and images were obtained using a Nikon Eclipse TS100. The number of cells that had undergone apoptosis was counted and quantified.

### *In vivo* studies

All animals were handled according to the approved protocol from IACUC at the University of Louisiana at Monroe. Athymic nude mice (Foxn1nu/Foxn1+, female, 4–5 weeks) were purchased from Harlan Laboratories (Indianapolis, IN) and maintained in the ULM School of Pharmacy vivarium. After acclimatization of animals to the local environment, estrogen (17β-estradiol) pellets (0.72 mg, 90-day release) were implanted subcutaneously in the mice, under anesthesia. Tumors were induced in mice as described previously [51]. Orthotopic injection of about three-million BT-474 cells suspended in the serum-free RPMI-1640 medium was done at the second left mammary fat pad [52]. Mice were observed every day for tumor formation, and the tumor diameter was measured with calipers, and the tumor volume was calculated [53]. When a tumor diameter of 3 mm was achieved, mice were randomly assigned to four groups of six. These groups contain negative control (vehicle control), positive control (lapatinib), and two treatment groups (compound 9 and 18). The mice were treated for 19 days. Compound 18 was administered near the base of the tumor at a dose of 4 mg/kg twice a week. Control groups received intratumor saline injections twice a week, and lapatinib was administered at 10 mg/kg once a week by intraperitoneal injection [39, 54]. The marker to end the experiments was based on tumor doubling time rather than tumor volume. Since tumor doubling time was 4 times in 19 days, experiments were stopped on the 19th day [55]. Tumor-bearing mice were sacrificed, and three tumors per group were harvested and maintained at –80°C and the remaining three tumors were fixed in paraffin blocks. Microsections (5 µm) of paraffin fixed tumors were stained with hemotoxylin and eosin (H&E) and observed for necrosis by a pathologist. All data are presented as the mean ± SE. Statistical differences were evaluated by Mann-Whitney *U* test as well as one-way ANOVA analysis of data from different groups, and the criterion for statistical significance was *p* < 0.05.

Western blot and PLA assay were carried out on tumor sections as described before [26]. Immunoblot analysis was performed to evaluate the levels of total HER2 and phosphorylated HER2. Tumor sections were deparaffinized using xylene and rehydrated with decreasing concentrations of ethanol. Antigen retrieval on the microsections of the breast tumors was done in a steaming sodium citrate buffer (10 mM, 0.05% Tween-20, pH 6.0) for 5 min. The tumor sections were then used to analyze inhibition of HER2:HER3 dimerization using PLA.

For evaluating the toxicity of the compound at the pharmacological dosage, 18 was administered intravenously at 6 mg/kg in athymic mice (Foxn1nu/Foxn1+, female, 4–5 weeks). 10 mice groups were used for obtaining samples for 10-time points (0, 15 min, 30 min, 45 min, 1 h, 2 h, 4 h, 6 h, 12 h, and 24 h). At each time point, the blood was withdrawn from the tail vein of mice and sacrificed after that. Serum was collected by centrifugation at 4000 rpm, and chilled methanol was added to precipitate the proteins in serum. It was vortexed and incubated on ice, followed by centrifugation at 5000 rpm for 10 min. Passing through SEP-PAK C18 column purified the supernatant and samples were analyzed using MS-MALDI. A freshly prepared solution of 20 (Table 2) in methanol (5 μM) was used as internal standard for calculating relative intensities of all time points. The organs were collected from the animals administered with 18 (24-h group) and from control mice and fixed in 10% formalin for 24 h and then embedded in paraffin. 5-μm sections were made for slides and stained using hematoxylin and eosin (H&E).

### NMR and modeling

Samples for NMR studies were prepared by dissolving 2 mg of the compounds in 650 µL of DMSO-d6. DSS was added as a reference. NMR data for the compounds were collected using a Bruker AVIII 500 MHz as well as a Varian VNMRS 700 MHz spectrometer. 1H NMR spectra were obtained for the samples at temperatures 25–35 °C and the spectra that showed good resolution were used for 1H 2D NMR data collected. TOCSY data were collected with 80 ms spin-lock time, and NOESY spectra were collected with 150 and 200 ms mixing time. A ROESY spectrum was also collected at 200 ms spin-lock time. Water peak in DMSO was suppressed during data collection. All the spectra were processed using Bruker and Varian software and converted into the SPARKY format. NMR data were analyzed using SPARKY[56]. Assignments of the spin systems in the compounds were analyzed using TOCSY and NOESY. NOESY intensity was converted into strong, medium, and weak with distance constraints 2–2.8 Å, 2.2–3.6 Å, 2.8–5 Å respectively. The structures of compounds were generated using Insight II software (BIOVIA, San Diego, CA). Distances generated from NOESY data were used as input for structures generated using Insight II. Upper and lower bound distances were applied with push and pull force of 50 kcal/mol. Å^2^. Structures were subjected to minimization after cyclization of the backbone. The structure of the compound generated was minimized with 100 steps of steepest descent method first before subjected to a simulated annealing procedure by carrying out the dynamics for 5 ps from 300 to 800 K and then decreasing the temperature back to 400 K in steps of 100 K. A 300 K dynamics was carried out for 20 ps and from the history file of 300 K dynamics structures were selected for every 100 steps. These structures were minimized using a conjugate gradient method until the rms derivative was 0.03 kcal/mol. Structures were verified for distances obtained from NOESY spectra. The minimized structures from 300 K dynamics was used as a representative structure for analysis.

### Docking

The crystal structure of HER2 protein extracellular domain was obtained from the Protein Data Bank (PDB ID 3N85) [57]. The structure of compound 18 and analogs was obtained as described above in the NMR and molecular modeling section. For docking, AUTODOCK software [58, 59] (Molecular Graphics Laboratory, La Jolla, CA) was used, and docking studies were performed as described before [27]. Briefly, a grid with a box was created with domain IV of HER2 at the center, and compound 18 and analogs were docked to HER2 protein domain IV with 10 million energy evaluations Calculations were performed on a Linux cluster using HPC at LSU Baton Rouge via the Louisiana Optical Network Initiative (LONI). From the results of docking calculations, 50 low-docking energy structures were analyzed. Structures with docking energy of < 2 kcal/mol from the lowest energy docked structure were used as representative structures for 18. Final structures were converted into PDB files and visualized using PyMOL Molecular Graphics System (Schrödinger, LLC Portland, OR).

### *In vitro* stability

One part of compound 18 stock solution (4.6 mM) was incubated with 9 parts of mouse serum to prepare 1000 µL of the mixture. For every time point, about 100 µL of the mixture was sampled, and 500 µL of cold methanol was added to it for serum proteins precipitation [60]. A similar method was used for sample extraction as described before. Briefly, samples were mixed by vortexing in cold condition and centrifuged at 5000 rpm for 10 min. The SEP-PAK C18 columns were used to separate the precipitated serum proteins. Methanol extract from SEP-PACK C18 column was collected and analyzed by mass spectrometry using Applied Biosystem LC/MS/MS Otrap instrument. A cyclic peptide compound 20, an analog of compound 5 (Table 2), was used as an internal standard for mass spectrometry analysis of extracted compound 18. The intensities of known concentration of internal standard were compared with compound 18 intensities for determination of relative quantification of compound 18.

## SUPPLEMENTARY MATERIALS FIGURES AND TABLE



## Abbreviations

ADCC: antibody-dependent cellular toxicity
BSA: bovine serum albumin
CTC-resin: Chlorotrityl Chloride resin
DAB: Diaminobenzidine
DAPI: 4′,6-diamidino-2-phenylindole
DCM: dichloromethane
DIEA: N,N-Diisopropylethylamine
DMF: Dimethylformamide
DMSO: dimethylsulfoxide
DSS: 4,4-dimethyl-4-silapentane-1-sulfonic acid
EGFR: Epidermal growth factor receptors
Fmoc: Fluorenylmethyloxycarbonyl chloride
HCTU: 1-[Bis(dimethylamino)methylen]-5-chlorobenzotriazolium 3-oxide hexafluorophosphate, *N,N,N′,N′*-Tetramethyl-*O*-(6-chloro-1*H*-benzotriazol-1-yl) uronium hexafluorophosphate
HER: human epidermal growth factor receptor
H&E: hemotoxylin and eosin
HRP: horse raddish peroxidase
LONI: Louisiana Optical Network Initiative
NSCLC: non-small cell lung cancers
MeOH: methanol
MS-MALDI: mass spectrometry-matrix assisted laser desorption ionization
NMP: N-Methyl-2-pyrrolidone
NOESY: Nuclear Overhauser enhancement and exchange spectroscopy
NRG-1: neuregulin-1
PDB: Protein Data Bank
p-HER2: phosphorylated HER2 protein
PLA: Proximity ligation assay
PPI: protein-protein interaction
PyAOP: 3-hydroxy-3H-1,2,3-triazolo[4,5-b]pyridinato-O)tri-1-pyrrolidinyl-phosphorus hexafluorophosphate
ROESY: rotating frame nuclear Overhauser enhancement spectroscopy
rTdT: recombinant Terminal Deoxynucleotidyl Transferase
SPR: Surface plasmon resonance
SSC: Saline-Sodium Citrate
TBST: Tris-buffered saline, 0.1% Tween 20
TFA: trifluoroacetic acid
THF: tetrahydrafuron
t-HER2: total HER2
TIPS: triisopropyl silane
TKI: tyrosine kinase inhibitors
TOCSY: total correlated spectroscopy
