# Supplementary material for: A peptidomimetic with a chiral switch is an inhibitor of epidermal growth factor receptor heterodimerization
Source: Oncotarget. 2017 Jul 5;8(43):74244–62. doi: 10.18632/oncotarget.19013 (PMC5650337; doi:10.18632/oncotarget.19013)
Supplement: Supplementary file 1 [file oncotarget-08-74244-s001.pdf]

A peptidomimetic with a chiral switch is an inhibitor of epidermal growth factor receptor heterodimerization

Supplementary Materials

Table 1: Analytical data for peptides

Code	Sequence	Calculated Molecular weight	Expt. Mass m/z	Purity by HPLC %
18	Cyclo(1,10)**P**^{1}**p**^{2}R^3(***R***-Anapa)^{4}F^{5}D^{6}D^{7}F^8(***R***-Anapa)^{9}R^{10}	1425.5893	1425.699	> 95
18-1	Cyclo(1,10)**P**^{1}**p**^{2}R^3(***S***-Anapa)^{4}F^{5}D^{6}D^{7}F^8(***R***-Anapa)^{9}R^{10}	1425.5893	1425.711	> 95
18-2	Cyclo(1,10)**P**^{1}**p**^{2}R^3(***R***-Anapa)^{4}F^{5}D^{6}D^{7}F^8(***S***-Anapa)^{9}R^{10}	1425.5893	1425.712	> 95
18-3	Cyclo(1,10)**P**^{1}**p**^{2}R^3(***S***-Anapa)^{4}F^{5}D^{6}D^{7}F^8(***S***-Anapa)^{9}R^{10}	1425.5893	1425.707	> 95
18-4	Cyclo(1,10)**p**^{1}**P**^{2}R^3(***S***-Anapa)^{4}F^{5}D^{6}D^{7}F^8(***S***-Anapa)^{9}R^{10}	1425.5893	1425.737	> 90
18-5	Cyclo(1,10)**p**^{1}**P**^{2}R^3(***R***-Anapa)^{4}F^{5}D^{6}D^{7}F^8(***R***-Anapa)^{9}R^{10}	1425.5893	1425.684	> 90
5	NH2-Arg-Anapa-Phe-OH*	518.60		> 95
9	NH2-Arg-Anapa-Phe-Asp-OH*	633.69		> 95
20	Cyclo(1,10)GPR-(Anapa)FDEFWR*	1388.52		95
21	Ac-f(Anapa)r-NH2*	559.65		> 95

*reported earlier in studies.

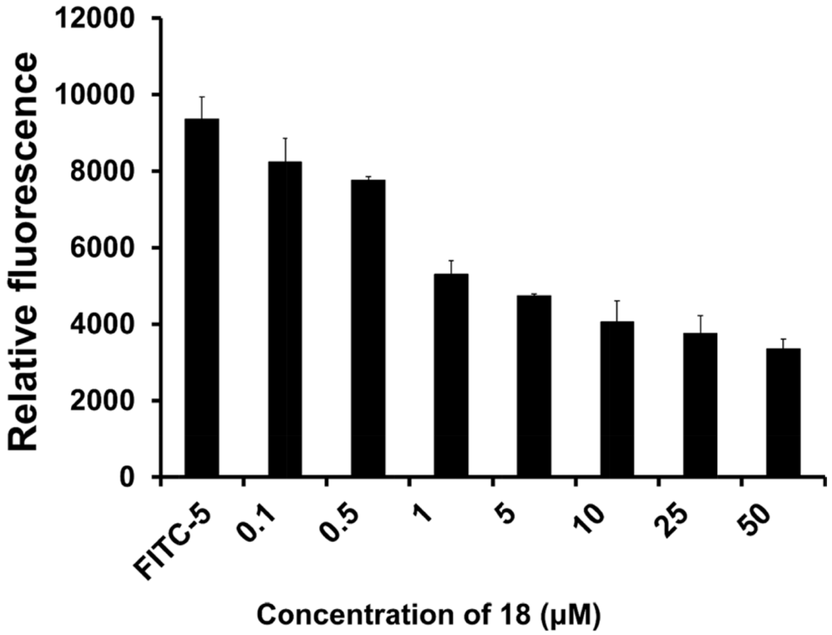

Supplementary Figure 1: Competitive binding of compound 18 to HER2 overexpressing BT-474 cells in the presence of FITC-5. FITC-5 is a compound which has been shown to bind to HER2 ECD ($P < 0.05$ for concentrations of the compound 18 at 1, 5, 10, 25 and 50 µM compared to value compared to FITC-5 alone).

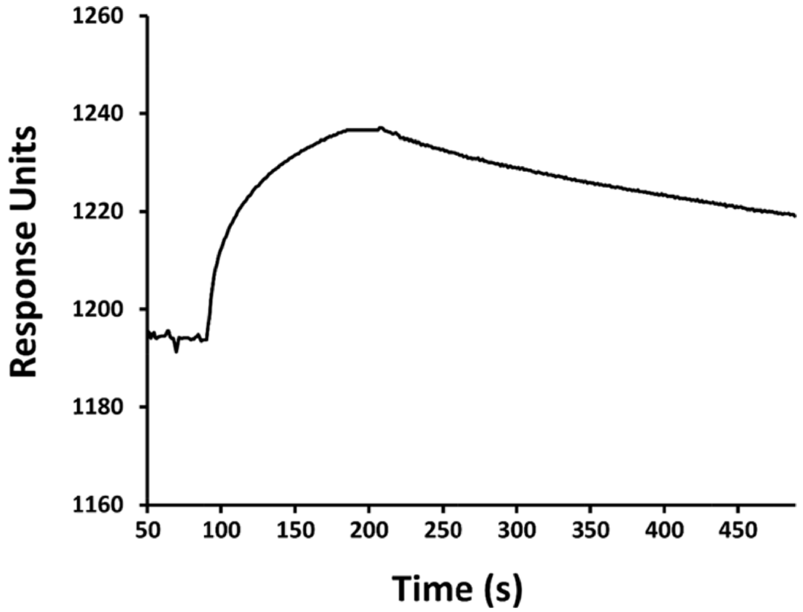

Supplementary Figure 2: Binding of antibody pertuzumab to HER2 protein ECD analyzed by SPR.

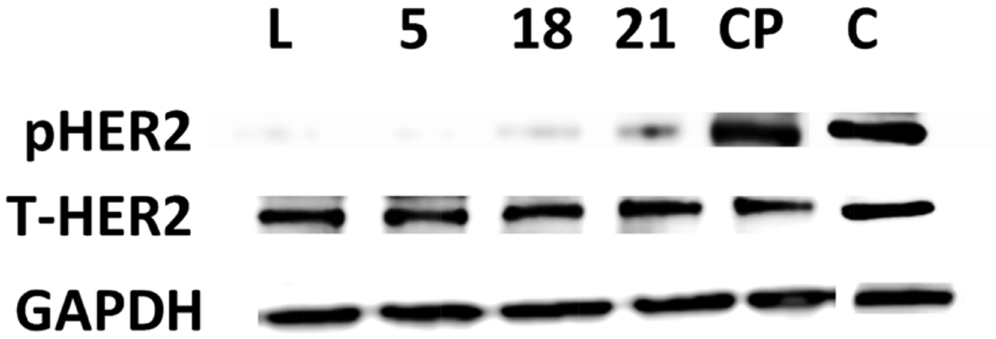

Supplementary Figure 3: Western blot analysis of compound 18 with total HER2 and phosphorylated HER2 and comparison with other compounds. L-lapatinib, CP-control peptide, C-control without any treatment of compounds.

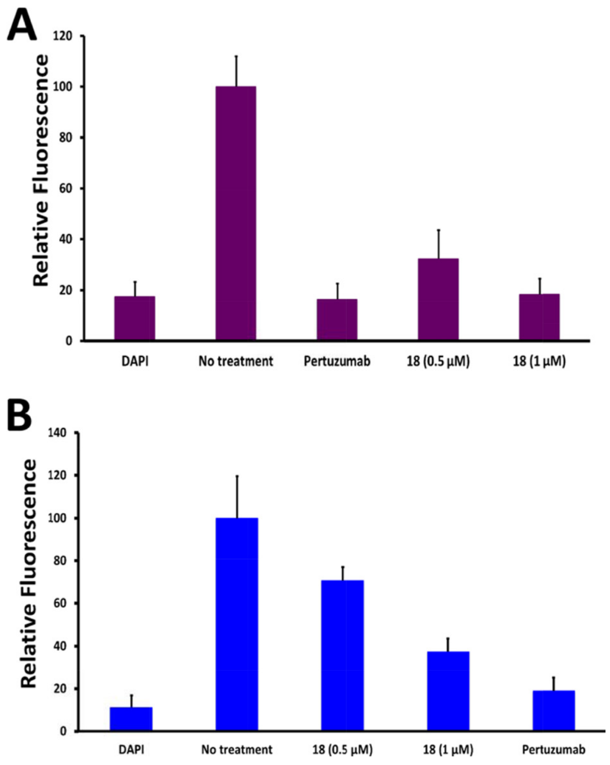

Supplementary Figure 4: Quantification of PLA assay and dose response of compound 18 on (**A**) HER2:HER3 inhibition and (**B**) EGFR:HER2 inhibition ($p < 0.05$ for compound 18 at 0.5 and 1 μM and pertuzumab when compared with control/no treatment).

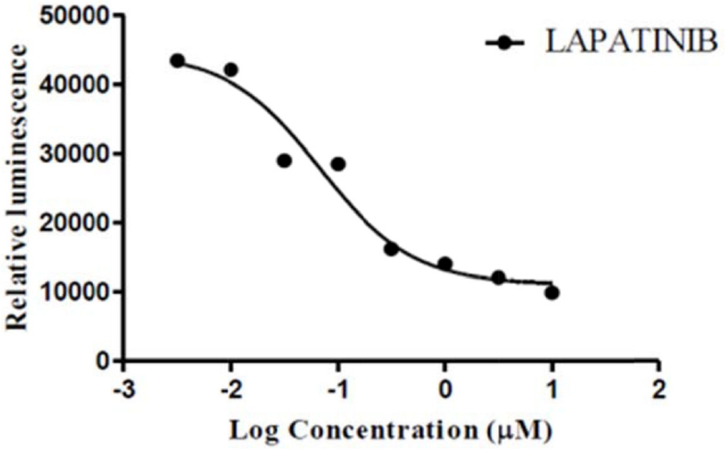

Supplementary Figure 5: Inhibition of heterodimerization of HER2-HER3 in transfected U2OS cells by lapatinib at different concentrations using PathHunter™ assay. Dose-response curve for inhibition of heterodimerization by lapatinib.

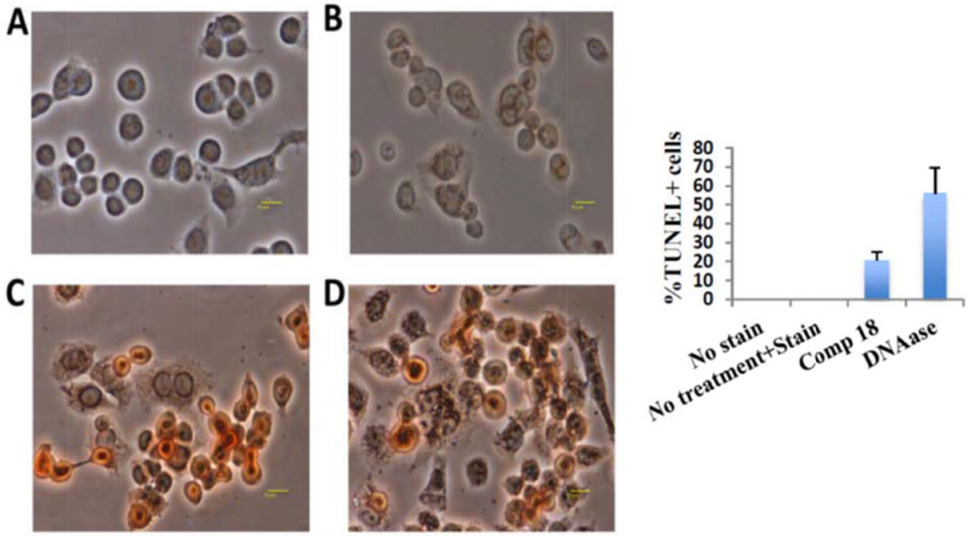

Supplementary Figure 6: Effect of compound 18 on SKBR-3 cells and apoptosis studied by TUNEL assay. Apoptotic effect of peptidomimetic compound on SKBR-3 cells using TUNEL assay. (**A**) Cells with no staining, (**B**) cells without any treatment (negative controls), (**C**) positive controls, cells with DNAase treatment, (**D**) treatment with compound 18 at 5 μM resulted in apoptosis. The quantification of results is shown on the right. *P* value < 0.01 for compound 18 compared to DNAse.

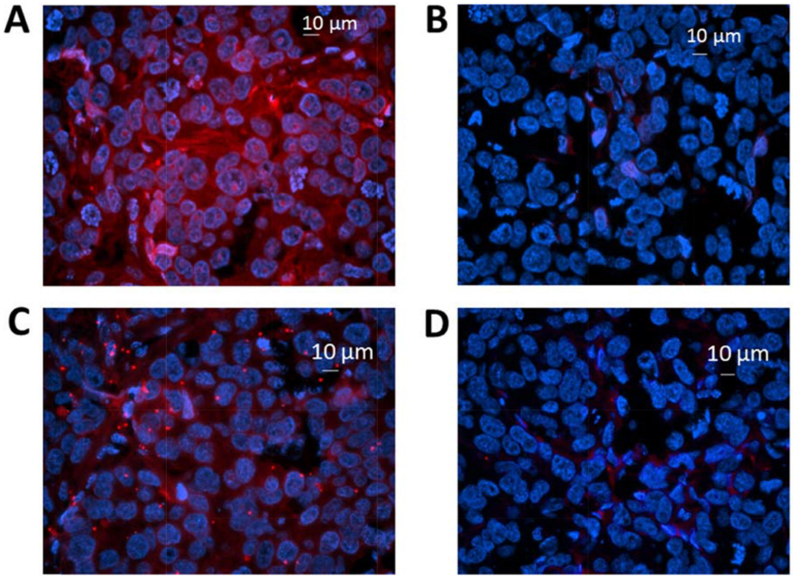

Supplementary Figure 7: Heterodimerization of EGFR and its inhibition by compound 18 studied using PLA assay in tumor section of samples derived from xenograft model of breast cancer. (**A**) sections from tumor without compound 18 treatment indicating PPI of HER2:HER3 shown by red fluorescence. (**B**) sections of tumor from animals without PLA probes. (**C**) sections of tumor that was treated with lapatinib. Notice the red fluorescence due to dimerization of HER2:HER3. Lapatinib is not a dimerization inhibitor. (**D**) sections of tumor with compound 18 treatment. Notice that there is no red fluorescence indicating inhibition of HER2:HER3 PPI (magnification 60×).

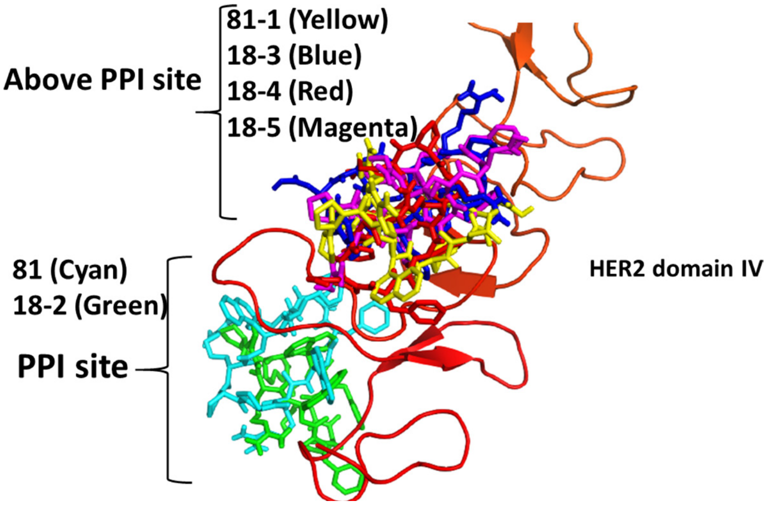

Supplementary Figure 8: Comparison of docking of diastereoisomers of compound 18 on HER2 protein domain IV. Note that compound 18 and 18-2 bind near the PPI site (refer to Supplementary Figure 8). Compounds 18-1,3,4 &5 bind above the PPI site. Thus, eventhough compounds 18-4 and 18-5 have docking energy comparable to that of compound 18, they will not inhibit PPI potentially and hence have relatively low antiproliferative activity compared to compound 18.

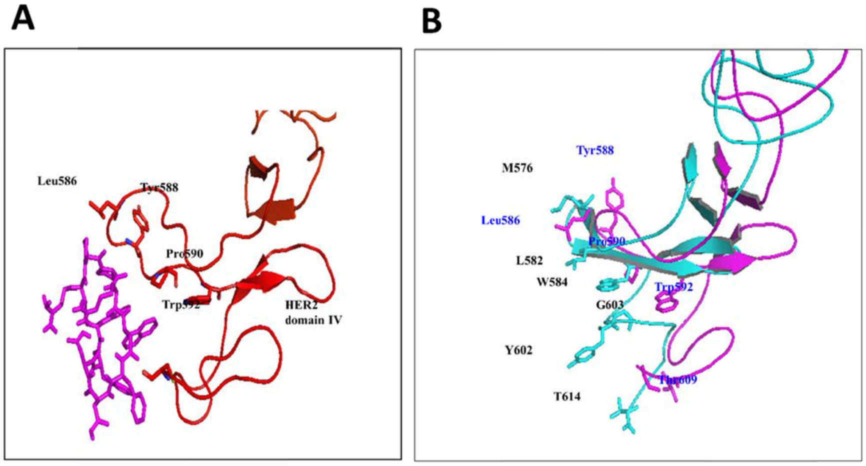

Supplementary Figure 9: (**A**) Lowest energy docked structures of compound 18 (−10.75 kcal/mol) with HER2 domain IV (PDB-3N85). (**B**) Overlapped crystal structures of EGFR (cyan) and HER2 (pink) at domain IV; residues important for protein-protein interactions at the epitopic structure of DIV of HER2 (blue) corresponding to the PPI surface of DIV of EGFR (black).

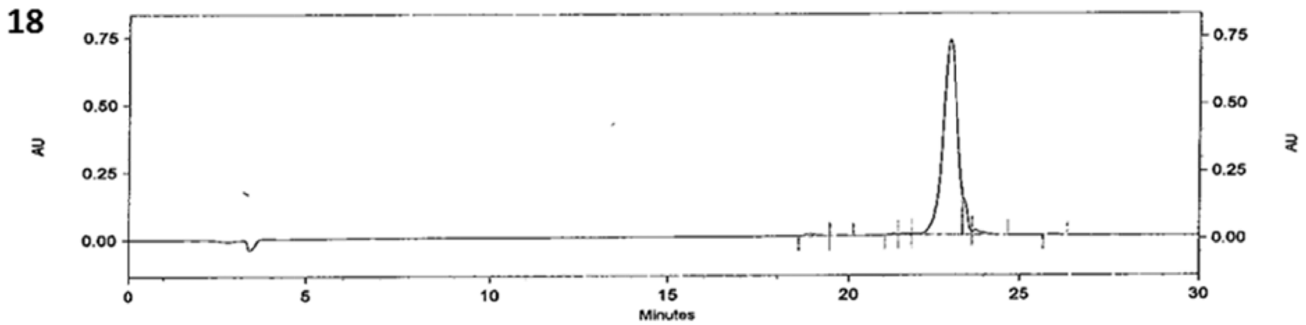

Supplementary Figure 10: HPLC of compound 18.

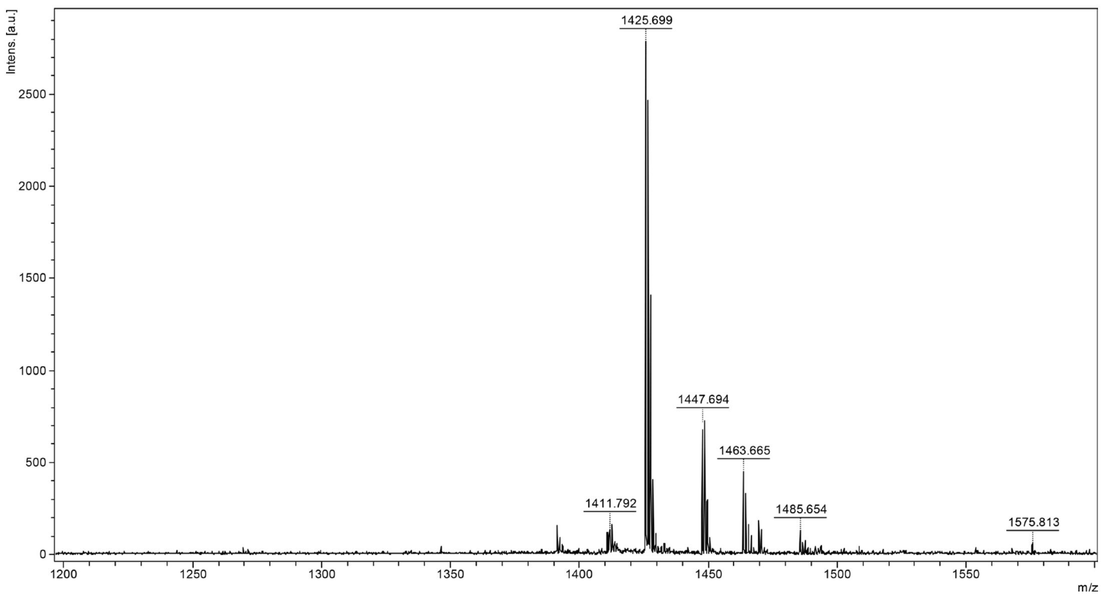

Supplementary Figure 11: High resolution mass spectra for compound 18.

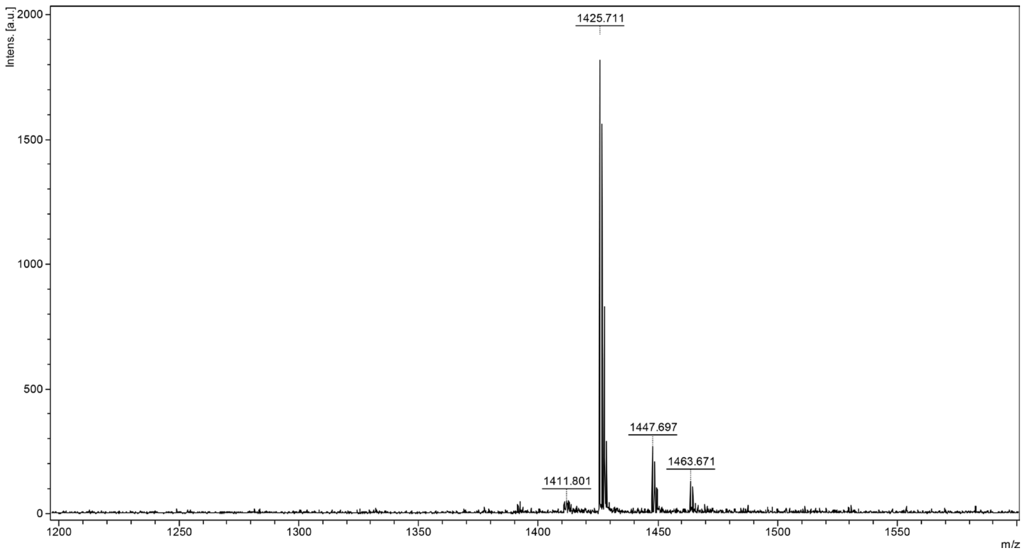

Supplementary Figure 12: High resolution mass spectrometry data for compound 18-1.

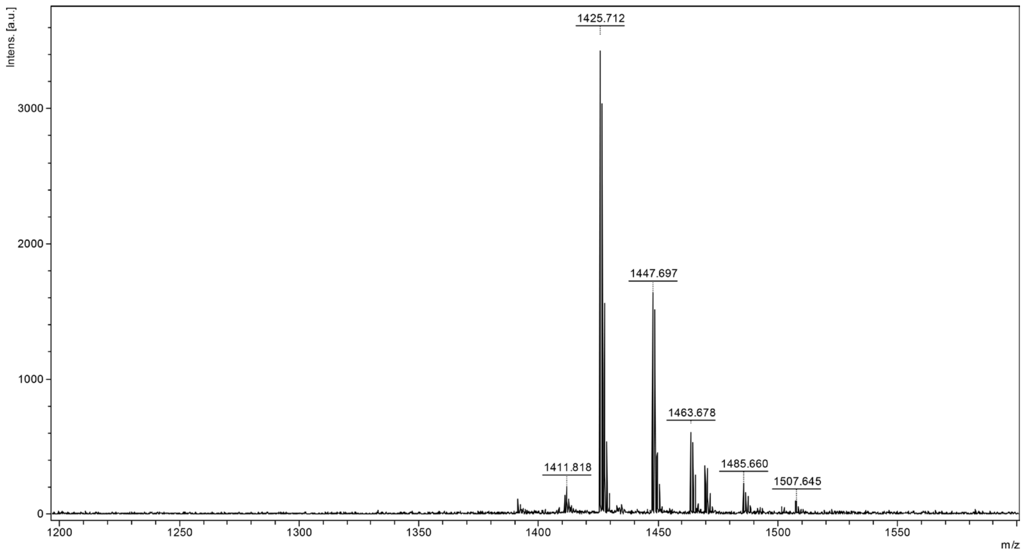

Supplementary Figure 13: High resolution mass spectrometry data for compound 18-2.

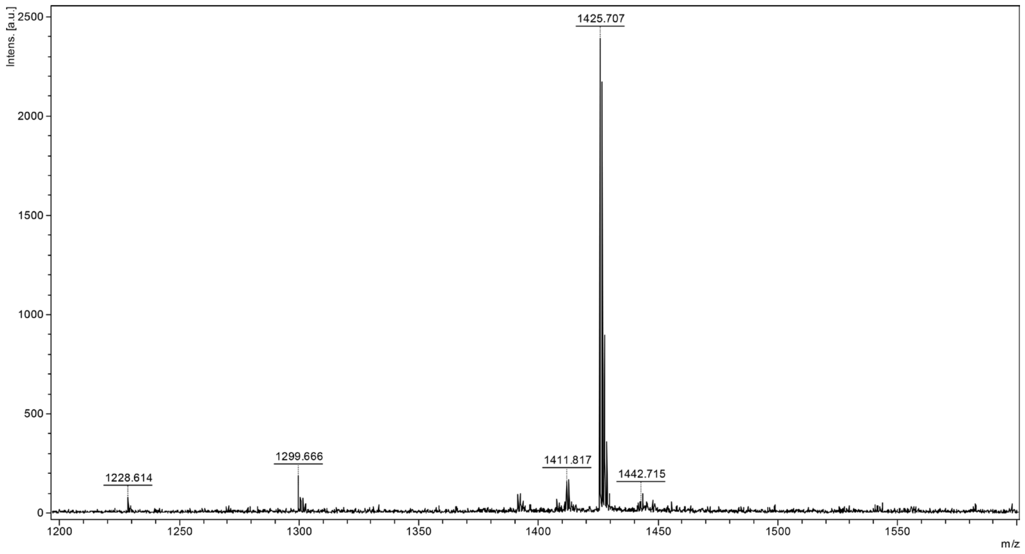

Supplementary Figure 14: High resolution mass spectrometry data for compound 18-3.

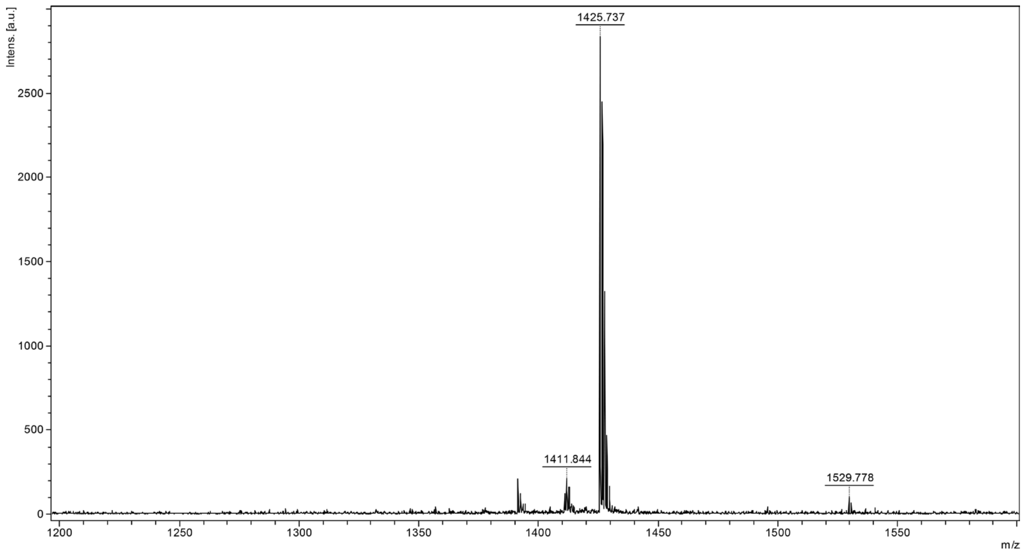

Supplementary Figure 15: High resolution mass spectrometry data for compound 18-4.

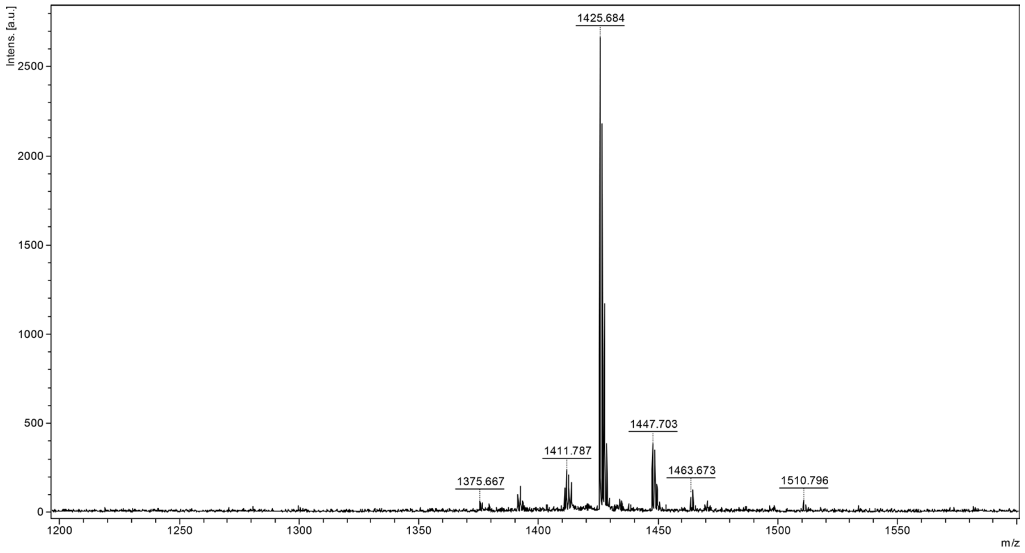

Supplementary Figure 16: High resolution mass spectrometry data for compound 18-5.

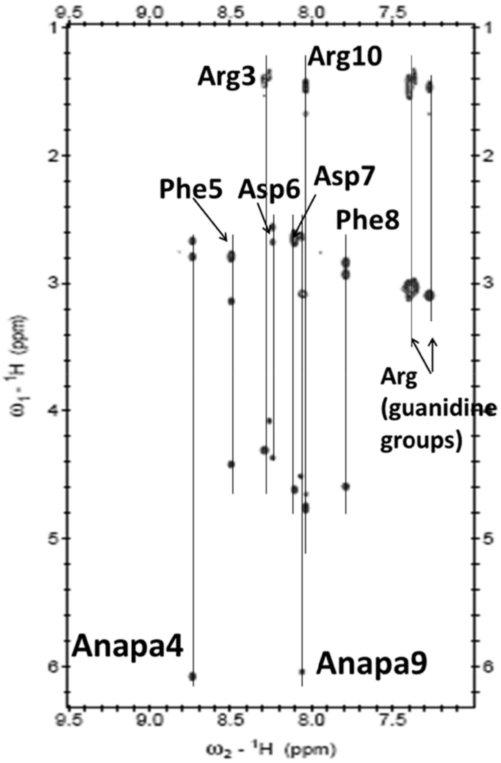

Supplementary Figure 17: 1H NMR 2D TOCSY spectra for compound 18 showing the fingerprint region. Connectivities within the amino acids/beta amino acids are shown.

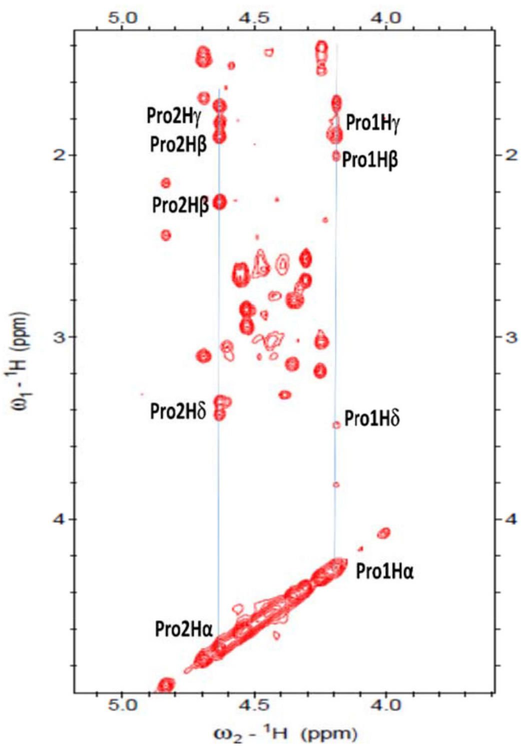

Supplementary Figure 18: 1H NMR 2D TOCSY spectra for compound 18 showing proline connectivities.

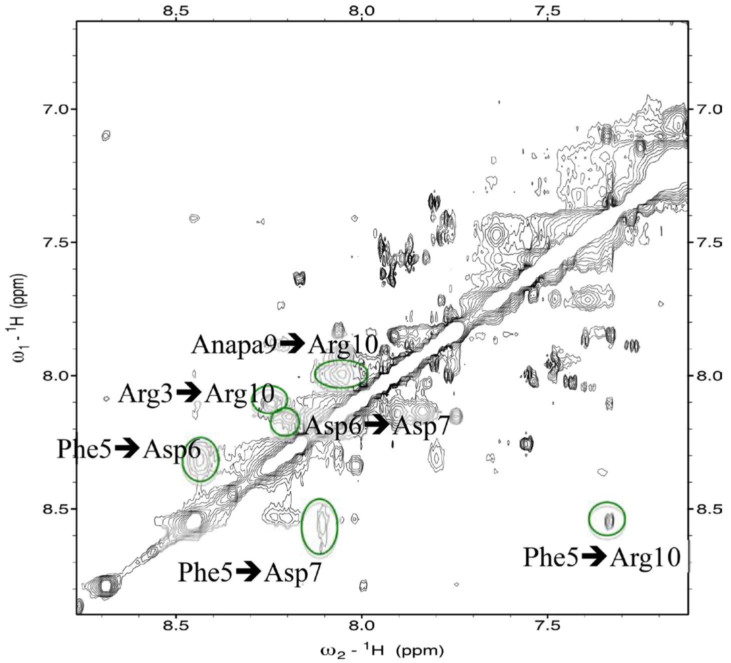

Supplementary Figure 19: 1H NMR 2D NOESY spectra for compound 18 showing NH-NH connectivities.

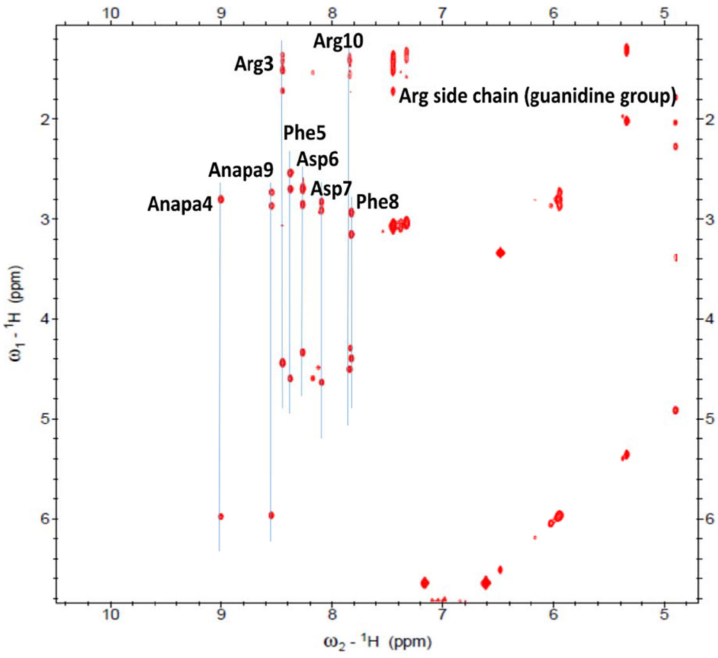

Supplementary Figure 20: 1H NMR 2D TOCSY spectra for compound 18-3 (diastereomer) showing the fingerprint region. Connectivities within the amino acids/beta amino acids are shown.
